# Effects of resistance-based training and polyphenol supplementation on physical function, metabolism, and inflammation in aging individuals

**DOI:** 10.1007/s11357-025-01839-8

**Published:** 2025-08-19

**Authors:** Mathias Flensted-Jensen, Cecilie Moe Weinreich, Ann-Sofie Kleis-Olsen, Filip Hansen, Nadia Stenner Skyggelund, Jeppe Rahbek Pii, Ryan Whitlock, Marie-Louise Brødsgaard Abrahamsen, Thomas Isbrandt Petersen, Anders Karlsen, Dace Reihmane, Flemming Dela

**Affiliations:** 1 Xlab, Department of Biomedical Sciences, Faculty of Health Sciences, University of Copenhagen, Blegdamsvej 3B, 2200 Copenhagen, Denmark; 2 Medical Faculty Mannheim, Heidelberg University, Heidelberg, Germany; 3 Asiros Nordic A/S, Sorø, Denmark; 4 Laboratory of Sports and Nutrition Research, Riga Stradiņš University, Riga, Latvia

**Keywords:** Aging, Inflammation, Exercise, Resistance training, Polyphenols

## Abstract

Aging is associated with declines in muscle mass, strength, aerobic capacity, and metabolic health, accompanied by increased low-grade inflammation. The purpose of this study was to assess the effects of 12 weeks of resistance training (RT) combined with minimal high-intensity interval training (HIIT), with or without polyphenol supplementation, on muscular, cardiovascular, metabolic, and inflammatory adaptations in healthy older adults. Forty-one men and women aged 55–70 years ingested either polyphenol supplementation or placebo for 30 days, then underwent 12 weeks of supervised RT combined with once-weekly HIIT, continuing polyphenol supplementation or placebo throughout. The training intervention increased whole-body and thigh lean mass, vastus lateralis cross-sectional area, type II fiber size, and muscle and functional strength (all *p* < 0.001). Maximal oxygen uptake significantly increased (*p* = 0.0001), accompanied by reductions in heart rate and plasma lactate during submaximal exercise, as well as increases in total blood volume and hemoglobin mass (*p* < 0.05). Training led to reductions in respiratory exchange ratio and plasma cortisol during exercise, which, together with decreased cholesterol levels and trends toward increased peak fat oxidation, may suggest enhanced substrate utilization. Polyphenols alone lowered cholesterol levels but had no other effects. Although no changes were observed in basal systemic or muscle inflammation, the exercise intervention attenuated the acute exercise-induced inflammatory responses of IL-10, IFN-γ, and TNF-α (*p* < 0.05). These results indicate that a combined RT and minimal HIIT program improves muscular, aerobic, and metabolic health, and may improve inflammatory regulation in aging adults.

**Supplementary Information:**

The online version contains supplementary material available at 10.1007/s11357-025-01839-8.

## Introduction

Aging is accompanied by a gradual decline in physiological function, including reductions in muscle mass [1], strength [1], and cardiorespiratory fitness [2], alongside an increased risk of chronic disease [3]. A key hallmark of aging is chronic low-grade inflammation, termed “inflammaging,” which links aging to functional decline and age-related diseases [4, 5]. As such, elevated levels of pro-inflammatory cytokines, such as tumor necrosis factor-alpha (TNF-α), interleukin-6 (IL-6), and C-reactive protein (CRP), have been associated with an increased risk of cardiovascular disease, insulin resistance, and neurodegenerative disorders [6]. Moreover, chronic inflammation has been linked to anabolic resistance [7] and muscle atrophy [8, 9], positioning it as a major contributor to the gradual loss of muscle mass, which ultimately reduces strength and functional independence in older adults. Beyond chronic inflammation, aging has also been shown to exaggerate the inflammatory response to physiological stressors, such as exercise, which may impair the beneficial adaptations following exercise [10–12].

Regular physical activity has been shown to counteract many of these negative effects of aging, with resistance training (RT) being especially beneficial for maintaining or even increasing muscle mass, strength, and metabolic health, in aging individuals [13]. However, its influence on metabolic flexibility and peak fat oxidation during exercise remains fairly underexplored. Overall, RT does seem to have positive effects on inflammation in elderly individuals [14], but a majority of current studies are confounded by either significant reductions in fat mass, lack of reporting on changes in body composition, or inclusion of subjects suffering from one or more conditions that may independently increase inflammation regardless of age (e.g., type 2 diabetes or cardiovascular disease) [14]. Thus, the anti-inflammatory effects of RT remain somewhat inconclusive. Additionally, high-intensity interval training (HIIT) has emerged as an effective strategy for improving cardiorespiratory fitness, body composition, and vascular function in older adults [15]. However, while the benefits of HIIT are well-documented, its effects on systemic inflammation in older adults remain less explored, and it is unclear whether combining resistance training with only a small dose of HIIT is sufficient to modulate systemic inflammation, fat metabolism, and cardiorespiratory fitness in older, inactive individuals.

Beyond exercise, nutritional strategies have been investigated for their potential to mitigate inflammation and enhance exercise adaptations. Polyphenol-rich foods and supplements, which contain bioactive compounds such as flavonoids, anthocyanins, and catechins, have been proposed to exert anti-inflammatory and antioxidant effects [16]. Some evidence suggests that polyphenol supplementation can enhance exercise recovery [17], increase fat oxidation [18], and improve cardiovascular health [19], yet these findings remain inconsistent and are largely based on animal and in vitro studies. Due to a lack of well-controlled clinical studies, the extent to which polyphenols can influence inflammation, muscle strength, and cardiovascular adaptations in older adults remains an open question. Besides modulating inflammation at rest, polyphenols may enhance exercise adaptations by modulating the exaggerated post-exercise inflammatory response in elderly individuals, yet this potential remains completely unexplored. Furthermore, it is not yet established whether pre-loading with polyphenols before engaging in a structured exercise program can enhance training adaptations or mitigate inflammatory responses over time.

This investigation aimed to determine whether RT, combined with a small dose of HIIT, and polyphenol supplementation influences basal and exercise-induced inflammation, muscle strength, fat oxidation, V̇O₂max, blood volume, and muscle fiber characteristics, in aging individuals. Understanding these relationships could have important implications for optimizing training and nutritional strategies aimed at promoting healthy aging and maintaining physical function in later life.

## Methods

### General study overview

All experimental days and training sessions took place at the Exercise Laboratory (Xlab) within the Institute of Biomedical Sciences, University of Copenhagen. Participants initially attended a screening session (A0). Those meeting the eligibility criteria were then randomized into either a polyphenol supplementation group or a placebo group. Following randomization, participants were provided with either a polyphenol supplement or a placebo, with instructions to take the supplement daily for a 30-day loading phase. Upon completion of the loading phase, participants returned to the laboratory for a follow-up testing day (A1), which was similar to the initial screening day. Two to 4 days after A1, participants attended an additional experimental session (B1). Following these baseline and loading assessments, participants began a 12-week RT-based intervention, continuing their assigned daily supplement. Post-intervention, participants returned for two final experimental days (A2 and B2), which replicated A1 and B1, respectively, to evaluate training-induced changes. The study was conducted in accordance with the Declaration of Helsinki and received approval from the Ethics Committee of the Capital Region of Denmark. The study was supported by a grant from the Center for Healthy Aging and from Asiros Nordic (Sorø, Denmark).

### Experimental days

On each experimental day, participants arrived at the research facility between 8:00 and 10:00 a.m. in a fasted state (having not eaten since 10:00 p.m. the previous evening), with instructions to refrain from exercise for 24 h prior and to avoid ingesting any supplements on the day of testing.

#### Initial screening day (A0)

Screening day assessments were conducted in a standardized sequence: (1) resting blood sample collection, (2) anthropometric measurements (weight, height, and body composition), (3) maximal fat oxidation testing, (4) maximal oxygen uptake (V̇O_2_max) assessment, and (5) strength assessments, which included handgrip strength, leg extension power, maximal voluntary contraction strength (MVC), rate of force development (RFD), and a 30-s sit-to-stand test. To minimize fatigue effects on strength assessments, participants were given a 60-min rest and provided a meal following the V̇O_2_max test.

#### Second experimental day (A1)

Protocols on day A1 mirrored those of A0, except no blood samples were taken, and a panoramic ultrasound measurement of the vastus lateralis cross-sectional area (CSA) was included following body composition measurements.

#### Third experimental day (B1)

Upon arrival, participants were positioned in a supine state, and two resting blood samples were collected at 10-min intervals. Following these samples, a muscle biopsy was taken from the right vastus lateralis. Subsequently, participants completed a 45-min moderate-intensity cycling test (65% V̇O_2_max), during which blood samples were drawn every 15 min and expired gases were continuously collected. Post-cycling, participants rested supine for 60 min, with blood samples collected at 15-min intervals. After the recovery period, blood volume and hemoglobin mass were assessed via carbon monoxide rebreathing. A meal was provided upon conclusion of these assessments, marking the end of the experimental session.

#### Post-intervention testing (days A2 and B2)

Following the training intervention, participants returned for days A2 and B2, which replicated the protocols of A1 and B1, respectively, to assess post-training outcomes. The A2 testing day was performed before the last or second-to-last training session, and the second biopsy day (B2) was completed exactly 2 days after the last training session.

### Participants

Participants were recruited primarily through social media advertisements and online research recruitment platforms. Inclusion criteria required participants to be between 55 and 70 years old, with a body mass index (BMI) of 20–30 kg/m^2^, weight stability (defined as less than ± 2 kg change within the past 6 months), and a relatively inactive lifestyle without recent training history. Exclusion criteria included elevated glycated hemoglobin (HbA1c > 42 mmol/mol), a history of heart failure (NYHA class II–IV), use of anticoagulant therapy, and a high fitness level (V̇O_2_max above the “moderate” range for their age group) [20]. Informed consent, both oral and written, was obtained prior to participation.

Following an initial phone screening, 63 participants were deemed eligible and invited for further evaluation. Of these, 51 met the full inclusion criteria and were enrolled in the study. Participants were randomly allocated to intervention groups using a computer-generated randomization sequence. The randomization was performed by an independent researcher not involved in participant recruitment, data collection, or analysis to ensure allocation concealment. Randomization was stratified for gender to ensure an equal gender ratio between groups. Between the screening and the first experimental session, 5 participants withdrew, and 5 participants discontinued during the intervention. Ultimately, 41 participants (21 men, and 20 women) completed the study. See supplementary data S1 for full flowchart of participant recruitment. Of the subjects that finished the intervention, 20 participants were allocated to the polyphenol group (10 men and 10 women, mean age 61 ± 4 years) and 21 were allocated to the placebo group (11 men and 10 women, mean age 62 ± 4 years). Baseline characteristics of participants are presented in Table 1.

**Table 1:** Baseline characteristics

	Placebo	Polyphenol	*p*-value
*N*	21	20	
Sex (m/w)	11/10	10/10	
Age (years)	62 ± 4	61 ± 4	0.434
Height (m)	1.74 ± 0.1	1.73 ± 0.1	0.723
Weight (kg)	78.9 ± 12.0	78.5 ± 7.7	0.903
BMI (kg/m^2^)	26.1 ± 3.3	26.2 ± 2.1	0.858
Fat percentage (%)	33.3 ± 8.3	34.3 ± 8.0	0.723
HbA1C (mmol/mol)	38 ± 2	37 ± 3	0.218
VO₂max (ml O₂/min/kg)	30 ± 5	30 ± 5	0.664

Subject characteristics at the screening visit (baseline) prior to supplement intake and training intervention. Data were analyzed using an unpaired *t*-test. Data are presented as means ± SD

### Polyphenol supplementation procedure

Participants assigned to the polyphenol supplementation group received a daily oral supplement containing a standardized dose of polyphenols (MitoActive, ASIROS NORDIC, Sorø, Denmark). The supplement was provided in liquid form, and participants were instructed to ingest 25 ml of the supplement daily, mixed with water. The assigned daily dose of the supplement contained approximately 700 mg of polyphenols derived from red- and blackcurrant. The placebo group received an oral supplement similar in taste, color, and macronutrient composition, in a similar container, but with no polyphenol content. Supplement and placebo were handed out by a person not involved in the study, and thus subjects and scientists were blinded in regard to supplement groups until all data was obtained.

The supplementation protocol included a 30-day loading phase before baseline testing and continued daily throughout the 12-week training intervention, providing consistent polyphenol exposure over the study duration. Participants were instructed to ingest the supplement in the morning, or as soon as possible during the day if they forgot it in the morning. Participants were further instructed to maintain their usual diet and physical activity levels outside of the structured training intervention to control for potential confounding variables. To monitor adherence to supplementation, participants received a checklist and were instructed to check a box and write down the date each time they ingested the supplement. According to the self-reported data, subjects had an average adherence rate of 98 ± 2% and no subjects reported an adherence rate below 90%.

### Polyphenol supplementation content

The polyphenol content and composition of the supplement were characterized by mass spectrometry, revealing a total polyphenol content of 0.98 ± 0.01% (gallic acid equivalents) and a diverse array of compounds, including flavonoids (anthocyanins, flavonols, and flavanols) and phenolic acids. LC–MS analysis identified key polyphenols such as caffeoylquinic acids, cyanidin derivatives, and myricetin, quercetin, and kaempferol glycosides, alongside ribetrils A–E. Cyanidin derivatives were among the most abundant anthocyanins, with a monomeric anthocyanin content of 0.005 ± 0.003% (cyanidin 3-glucoside equivalents). See supplementary data (S8) for full mass spectrometry analysis.

### Training intervention

The 12-week training intervention consisted of three weekly supervised sessions, with 1 day of rest between sessions. Sessions started with a 5-min warm-up on a cycle ergometer, and each exercise started with a warm-up set. Two sessions focused on full-body machine-based (Technogym, Cesena, Italy) RT, while the third session consisted of lower limb resistance exercises followed by HIIT. RT included the following exercises: leg press, leg extension, leg curl, lat pulldown, seated rows, chest press, and shoulder press. Lower limb exercises progressed from 3 sets of 12 repetition max (RM) (weeks 1–3) to 3 sets of 10 RM (weeks 3–6), then 4 sets of 8 RM (weeks 6–9), and 4 sets of 6 RM (weeks 9–12). Upper body exercises were performed at 15 RM (weeks 1–4), 12 RM (weeks 4–8), and 10 RM (weeks 8–12). The HIIT was performed on a BikeErg cycle ergometer (Concept2, Morrisville, Vermont, USA) and began with two warm-up sets of 60 s at 90% of V̇O_2_max, defined as 90% of the resistance that elicited V̇O_2_max during the V̇O_2_max test, with 60-s breaks in between sets. Warm-up sets were followed by 5–7 working sets of 60 s, each separated by 60-s rest intervals. During weeks 1–4, the warm-up was followed by three sets of 60 s at 100% V̇O_2_max and two sets of 60 s performed all-out. In weeks 5–8, participants completed four sets at 105% V̇O_2_max and two all-out sets. During weeks 9–12, the protocol progressed to four sets at 110% V̇O_2_max and three all-out sets. To monitor the training intensity and ensure subjects trained at the correct intensity, power output and peak heart rate were recorded for each interval at all training sessions (see supplementary data S4 and S5).

### Body composition

Body composition and thigh lean mass were determined by dual-energy X-ray absorptiometry (DXA) (Lunar iDXA, G&E Medical Systems Lunar, Wisconsin, USA). Thigh lean mass was determined from DEXA images by defining regions of interest using the DEXA software. Lines were drawn between the knee joint line, defined as the distal border, and through the femoral neck proximal of the greater trochanter, defined as the proximal border. Regions of interest (ROIs) for each individual were saved and copied to images of the same individual. The investigator was blinded to the condition and timepoint of the scans. Vastus lateralis (VL) CSA was determined by panoramic ultrasound imaging (Vivid Iq, GE Healthcare, Chicago, IL). Subjects were scanned at the middle of the thigh, defined as halfway of the distance from the greater trochanter to the lateral epicondyle of the femur. Images were analyzed by an investigator who was blinded to the subject group and timepoint.

### Strength measurements

Quadriceps MVC was assessed during maximal isometric knee extensor efforts performed at a knee joint angle of 70° (0° = full extension) using an isokinetic dynamometer (KinCom 125AP, Chattecx Corp., Chattanooga, USA). The peak RFD was derived from the isometric MVC.

Maximal leg extension power (LEP) was assessed using a Nottingham Leg Extension Power Rig (Medical Engineering Unit, University of Nottingham Medical School, Nottingham, UK). Participants, seated with a 15° knee angle, pushed a footplate connected to a flywheel as forcefully and quickly as possible. The flywheel’s maximum speed was used to calculate the average lower limb extensor power.

Handgrip strength was measured using a Jamar dynamometer (Sammons Preston Rolyan, Chicago, Illinois, USA) over three trials, each separated by 1-min rest. Participants were seated upright with their arm at a 90° elbow angle and the best trial for each arm was recorded.

A 30-s sit-to-stand test (STS) was performed on a standardized armless chair, and subjects were instructed to stand up and sit down as many times as possible for 30 s, starting from the sitting position with their arms crossed over their chest and ending with full hip extension.

A 3-repetition max (3RM) test was performed for the leg extension exercise at the beginning of a training session, once every 4 weeks throughout the training intervention. Subjects started with two warm-up sets of approximately 50% and 80% of their estimated 3RM and were then instructed to lift the selected weight three times. If subjects succeeded, the weight was increased until the subjects failed to complete three repetitions with full range of motion.

### Cardiorespiratory fitness and fat oxidation

Participants underwent two types of exercise tests on a bicycle ergometer (COSMED, E100 ergoline, Germany): a maximal fat oxidation (MFO) test and a maximal oxygen consumption (V̇O_2_max) test, separated by a 5-min rest. Pulmonary gas exchange (V̇O_2_ and CO₂) was measured and analyzed using a mixing chamber system (CPET, COSMED, Italy). The MFO test began with 5 min of rest, followed by a 5-min warm-up at 30 W. Subsequently, the workload increased by 20 W every 3 min until a respiratory exchange ratio (RER) of 1.0 was achieved. MFO values were calculated based on established methods [21]. The V̇O_2_max test started at 30 W, with workload increasing by 20 W every minute until participants reached voluntary exhaustion. V̇O_2_max attainment was confirmed if at least two of the following criteria were met: RER > 1.15, a plateau in V̇O_2_ or heart rate despite increasing workload, or failure to maintain a cadence above 60 rpm. All participants satisfied at least two of these criteria.

On experimental days B1 and B2, subjects performed a 45-min cycling test, at an intensity corresponding to 65% of the resistance that elicited V̇O_2_max during the V̇O_2_max test. Pulmonary gas exchange was recorded during the test (CPET, COSMED, Italy). On day B1, during the initial 15 min of the test, the resistance was adjusted until the target V̇O_2_ of 65% V̇O_2_max was met, and the remaining 30 min was performed at that intensity with no changes. The resistance at specific time points was written down and replicated on day B2.

### Blood analyses

Blood samples were collected at rest and in the fasted state on the screening day and days B1 and B2. Additional samples were taken every 15 min during the 45-min cycling test and the following 60-min recovery period. All samples were collected in tubes containing various anticoagulants (dipotassium (K₂) EDTA/aprotinin; tripotassium (K₃) EDTA/aprotinin; lithium heparin; sodium fluoride (NaF)/potassium oxalate). The samples were centrifuged (ROTINA 380R, Axeb Lab Solutions) at 4000 g for 10 min at 4 °C to separate plasma, which was then transferred into Eppendorf tubes and stored at − 80 °C until analysis. Plasma samples were analyzed for metabolites (ketones, glucose, lactate, FFA, triglycerides, glycerol), CRP, cortisol, lipoproteins (HDL, LDL, and total cholesterol), and insulin using a COBAS e601 system with electrochemiluminescence immunoassay (ECLIA).

Custom human 10-plex kits using MULTI-ARRAY electrochemiluminescence detection technology (MesoScale Discovery, Gaithersburg, MD) were used for the quantitative evaluation of the following ten different human cytokine and chemokine analytes in plasma samples: IL6, TNFα, IL1β, IL8, IL10, IL4, IL18, IFN-γ, MCP1, and IL4. Plasma samples were measured, following the manufacturer’s instructions, in duplicate using 25 µL of undiluted plasma per replicate. Analyte concentrations (pg/mL) from both runs of each analyte were averaged for analysis. Cytokine values below the lower limit of detection or above the upper limit of detection were estimated by the multiplex assay using extrapolation from the standard curve. Values above or below the fit curve range were reported missing by the assay. HbA1c was determined at the screening only, using a DCA Vantage Analyzer (Siemens Healthineers, Forchheim, Germany).

### Blood volume and hemoglobin mass

Blood volume and hemoglobin mass were determined by carbon monoxide (CO) rebreathing, before and after the training intervention on experimental days B1 and B2, at the end of the 60-min recovering period following exercise. The CO rebreathing technique has been described in detail previously [22]. In brief, participants rested in a supine position with their legs slightly elevated while an initial blood sample was drawn. This sample was analyzed in duplicate to determine carboxyhemoglobin levels (%HbCO) and hemoglobin concentration using an ABL800 analyzer (Radiometer). Hematocrit was measured using the micro-method, which involved centrifugation for 4 min at 13,500 rpm. Subsequently, each participant was connected to a semi-automated blood volume analyzer (Detalo Performance, Detalo Health, Denmark). This device delivered a bolus of 99.997% chemically pure carbon monoxide (1.0 mL per kg of body weight; AGA, Lidingö) into a closed rebreathing system. Participants rebreathed the mixture, which included 100% oxygen, for 10 min. A second blood sample was collected at the end of the 10 min and analyzed in duplicate for %HbCO. Total red blood cell volume (RBCV), plasma volume (PV), and blood volume (BV) were calculated based on total hemoglobin mass, hemoglobin concentration, and hematocrit values.

### Muscle biopsies

Muscle biopsies were obtained on days B1 and B2 from m. vastus lateralis with a Bergström needle with manual suction at rest. Selected sections of muscle tissue were embedded in OCT embedding matrix (CellPath, Newtown, United Kingdom), frozen in isopentane pre-cooled by liquid nitrogen, and stored at − 80 °C. The remaining tissue was snap frozen in liquid nitrogen and stored at − 80 °C for later analyses.

### Immunofluorescence

Muscle samples were prepared for immunofluorescence (IF) by cutting 10-µm sections in the cross-sectional plane with a cryostat at − 20 °C. The sections were placed on glass slides and allowed to dry before being stored at − 80 °C. Before staining, fixation was performed for 5 min in 4% PFA (Avantorsciences, Cat. No.22023). Incubation with primary antibodies at 4 °C overnight was followed by incubation with secondary antibody incubation at room temperature for 45 min in the dark. Sections were then mounted under coverslips with a mounting medium (Invitrogen™ ProLong™ Gold Antifade Mountant with DNA Stain DAPI, Fischerscientific), dried for 2 days at room temperature in the dark, and stored at − 20 °C until imaging. In between all steps, sections were washed 3 × 5 min in 1 × PBS. All antibodies were diluted in 1% BSA IgGfree (Merck, Cat. no. A9085) in 1 × PBS. Primary antibodies (Developmental Studies Hybridoma Bank (DSHB)) are as follows: MyHC-I (A4.951, 1:50, Mouse IgG1), MyHC-IIX (6H1, 1:50, Mouse IgM), Laminin Gamma-1 (2E8, 1:50, Mouse IgG2a). A4.951, 6H1, and 2E8 were deposited to the DSHB by Blau, H.M., Lucas, C. and Engvall, E.S respectively. Secondary antibodies (Goat anti-Mouse, Thermofischer) are as follows: 488 (Green, A-21121, 1:500, IgG1), 568 (Red, A-21043, 1:500, IgM), 647 (Far-red, A-21241, 1:500, IgG2a).

### Microscopy and analysis of cross-sectional area

Imaging was performed with a Zeiss Axioscan Z1 fluorescent microscope, using a 10 × objective. In all biopsies, 4-channel images were taken of the whole biopsy, and stitched into one large composite image by the software. The images were then imported into ImageJ (version 1.53n, National Institutes of Health, Bethesda, MD, USA), and an automatic delineation of the fibers were performed with a modified version of a macro previously described in detail in [23]. The automated analysis was followed by a manual approval step by the same investigator who was blinded during the analysis. The manual step included verifying the fibertyping, excluding the fibers in the perimeter of the biopsies, excluding regions not cut in the cross-sectional plane, and excluding fibers and regions with poor staining quality where the automated delineation failed to detect the fiber membranes correctly. The signal in the MyHC-I channel was used to distinguish between type I and type II fibers. On average, 246 ± 117 type I fibers and 235 ± 143 type II fibers were included in the analysis.

### Gene expression analysis

Gene expression analysis was performed in muscle tissue samples from before and after the training intervention from both groups. Due to insufficient amount of tissue, the analysis included 11 subjects from the polyphenol group and 15 subjects from the placebo group. Total RNA was isolated from tissues with chloroform extraction using Invitrogen Trizol TM Reagent (ThermoFisher Scientific, Australia) following the instructions provided by the manufacturer. After extraction, RNA concentration was measured using a Qubit (Thermo Fisher Scientific, USA). A total of 500 ng of RNA was converted into cDNA using Quantitect Reverse Transcription (Qiagen, Germany), following the instructions from the manufacturer. Quantitative PCR (qPCR) was performed using the QuantiNova SYBR Green PCR Kit (Qiagen, Germany). Quantification of mRNA expression was performed according to the delta‐delta Ct method. All results were normalized to Actin β as the housekeeping gene. The analysis included the following genes: Toll-like receptor 4 (TLR4), monocyte chemoattractant protein-1 (MCP-1), and nuclear factor kappa-light-chain-enhancer of activated B cells (NF-κB). For primer sequences, see Supplementary Data S6.

### Statistical analysis

Data was analyzed and figures created using Graphpad Prism 10.4.0 (Dotmatics, San Diego, CA, USA). Statistical analyses of parameters measured across the intervention phases were performed with a two-way analysis of variance (ANOVA) or a mixed model in the case of missing data, with time as the within-subject factor and group as the between-subject factor. In case of an interaction effect, post hoc tests were performed and the Šidák method was used to adjust for multiple comparisons. For measurements that included tests both at baseline (SCR), after 1 month of supplementation (before training) (PRE) and after training (POST), analyses were carried out comparing SCR to PRE and then comparing PRE to POST, to investigate and differentiate the effect of supplementation alone and training + supplementation. Differences between groups in baseline characteristics were determined by an unpaired *t*-test. All missing data were assumed to be missing at random. Data was tested for normality distribution using the Shapiro-Wilks test, QQ plots, and residual plots. The data were normally distributed and are presented as mean values ± standard deviation (SD). *p*-values < 0.05 were considered significant.

## Results

### Sex differences at baseline

At baseline, the male participants displayed higher lean mass, both total and relative to total body mass (*p* < 0.0001 for both), lower fat mass (*p* = 0.004) and fat percentage (*p* < 0.0001), higher visceral fat mass (*p* = 0.03), and higher bone mineral density (*p* < 0.0001), compared to female participants. Male participants had larger muscle fiber CSA for both type I (*p* = 0.001) and type II muscle fibers (*p* = 0.0002). Male participants had higher total MVC (*p* < 0.0001) with a tendency towards higher MVC relative to lean mass (*p* = 0.08), higher handgrip strength both total (*p* < 0.0001) and relative to lean mass (*p* = 0.03), and higher total (*p* < 0.0001) but not relative (*p* = 0.2) LEP. There were no differences in STS test reps, but female participants performed more reps relative to their lean mass (*p* < 0.0001). Male participants had a higher V̇O_2_max, both total (*p* < 0.0001) and relative to bodyweight (*p* = 0.0003). Female participants had lower total resting fat oxidation rates (*p* = 0.004), but higher resting (*p* < 0.0001) and maximal (*p* = 0.01) fat oxidation rates when normalized to lean mass. Male participants had higher blood volume and hemoglobin mass (*p* < 0.0001 for both), both total and when normalized to body weight (*p* < 0.005 for both), while no differences were present when normalized to lean mass. Female participants displayed lower plasma IL-6 levels (*p* < 0.05), but no other plasma inflammatory markers or metabolites were different.

### Body composition

There were no differences in age, body composition, or HbA1C levels between groups at baseline (Table 1). Bodyweight, BMI, total fat mass, visceral fat, and bone mineral density did not change over the course of the training intervention (Table 2), and no body composition measurements changed during the loading phase (from SCR to PRE). Following the training intervention, whole-body lean mass increased by 1.7 ± 2.2% in the placebo group and by 1.7 ± 2.1% in the polyphenol group (time effect: *p* < 0.0001), with no differences between groups (time × group effect: *p* = 0.946) (Table 2). Right thigh lean mass increased by 3.7 ± 2.6% in the placebo group and by 3.6 ± 2.4% in the polyphenol group (time effect: *p* < 0.0001) post-training, with no differences between groups (time × group effect: *p* = 0.673) (Table 2). Whole-body fat percentage decreased by 0.6 ± 1.4 percentage points in the placebo group and by 0.9 ± 2.6 percentage points in the polyphenol group (time effect: *p* = 0.039), following the training intervention, with no differences between groups (time × group effect: *p* = 0.639) (Table 2). VL muscle CSA increased by 6.9 ± 13.4% in the placebo group and by 10.8 ± 12% in the polyphenol group (time effect: *p* = 0.0004), with no differences between groups (time × group effect: *p* = 0.495) (Table 2).

**Table 2:** Body composition and muscle characteristics

	Placebo (*n* = 21)			Polyphenol (*n* = 20)			*p*-value SCR to PRE		*p*-value PRE to POST	
	SCR	PRE	POST	SCR	PRE	POST	Time	Time × group	Time	Time × group
Weight (kg)	78.9 ± 12.0	79.1 ± 11.8	79.1 ± 11.8	78.5 ± 7.7	78.6 ± 7.8	78.5 ± 7.7	0.416	0.581	0.561	0.476
BMI (kg/m^2^)	26.1 ± 3.3	26.1 ± 3.3	26.3 ± 3.4	26.2 ± 2.1	26.2 ± 3.3	26.2 ± 1.8	0.398	0.668	0.646	0.527
Whole-body fat mass (kg)	26.2 ± 7.7	26.4 ± 7.6	26.0 ± 8.0	26.5 ± 6.9	26.7 ± 6.8	25.9 ± 6.2	0.180	0.960	0.077	0.463
Fat percentage (%)	33.3 ± 8.3	33.4 ± 8.3	32.9 ± 8.7	34.3 ± 8.0	34.2 ± 7.9	33.3 ± 7	0.987	0.659	**0.039**	0.639
Visceral adipose tissue (g)	1266 ± 742	1311 ± 732	1280 ± 792	1209 ± 676	1241 ± 758	1165 ± 658	0.136	0.792	0.143	0.530
Bone mineral density (g/cm^2^)	1.205 ± 0.2	1.203 ± 0.2	1.203 ± 0.2	1.162 ± 0.1	1.164 ± 0.1	1.165 ± 0.1	0.992	0.697	0.929	0.692
Whole-body lean mass (kg)	52.5 ± 9.8	52.6 ± 10.1	53.5 ± 10.4	51.9 ± 8.1	51.9 ± 8.0	52.8 ± 8.7	0.902	0.473	** < 0.0001**	0.946
Right thigh lean mass (kg)	4.4 ± 0.9	4.4 ± 0.9	4.6 ± 0.9	4.4 ± 0.7	4.4 ± 0.6	4.6 ± 0.7	0.311	0.344	** < 0.0001**	0.673
Vastus lateralis CSA (cm^2^)	-	17.9 ± 5.5	19.1 ± 5.8	-	15.4 ± 3.5	17.1 ± 4.6	-	-	**0.0004**	0.495
Type II fiber CSA (µm^2^)	-	3469 ± 1429	4036 ± 1502	-	3636 ± 1528	4178 ± 1856	-	-	**0.003**	0.798
Type I fiber CSA (µm^2^)	-	4229 ± 1238	4338 ± 1225	-	4021 ± 1044	4059 ± 926	-	-	0.589	0.793
Type II fiber area (%)	-	48.7 ± 13.4	50.2 ± 14.9	-	47.1 ± 12.4	50.0 ± 10.2	-	-	0.235	0.492
Type I fiber area (%)	-	51.3 ± 13.4	49.8 ± 14.9	-	52.9 ± 12.4	50.0 ± 10.2	-	-	0.235	0.492

Body composition measurements and muscle characteristics determined at the screening visit (SCR), after 1 month of supplement intake (PRE) and after 12 weeks of training with continuous supplementation (POST). Weight, BMI, whole-body fat mass, fat percentage, visceral adipose tissue, bone mineral density, whole-body and right thigh lean mass determined by DXA. Vastus lateralis CSA determine by panoramic ultrasound. Types I and II fiber size and distribution determined by immunohistochemistry. Data were analyzed using a two-way ANOVA. *p*-value < 0.05 was considered significant and is indicated in bold. Data are presented as means ± SD

Body composition measurements and muscle characteristics determined at the screening visit (SCR), after 1 month of supplement intake (PRE) and after 12 weeks of training with continuous supplementation (POST). Weight, BMI, whole-body fat mass, fat percentage, visceral adipose tissue, bone mineral density, whole-body and right thigh lean mass determined by DXA. Vastus lateralis CSA determine by panoramic ultrasound. Types I and II fiber size and distribution determined by immunohistochemistry. Data were analyzed using a two-way ANOVA. *p*-value < 0.05 was considered significant and is indicated in bold. Data are presented as means ± SD.

The training intervention resulted in a significant increase in type II muscle fiber CSA from 3469 ± 1429 to 4036 ± 1502 µm^2^ in the placebo group, and from 3636 ± 1528 to 4178 ± 1856 µm^2^ in the polyphenol group (time effect: *p* = 0.0025), with no differences between groups (time × group effect: *p* = 0.798) (Table 2). The training intervention did not affect type I fiber size nor distribution of type I and type II fibers in either group (Table 2).

### Strength

**Fig. 1:**
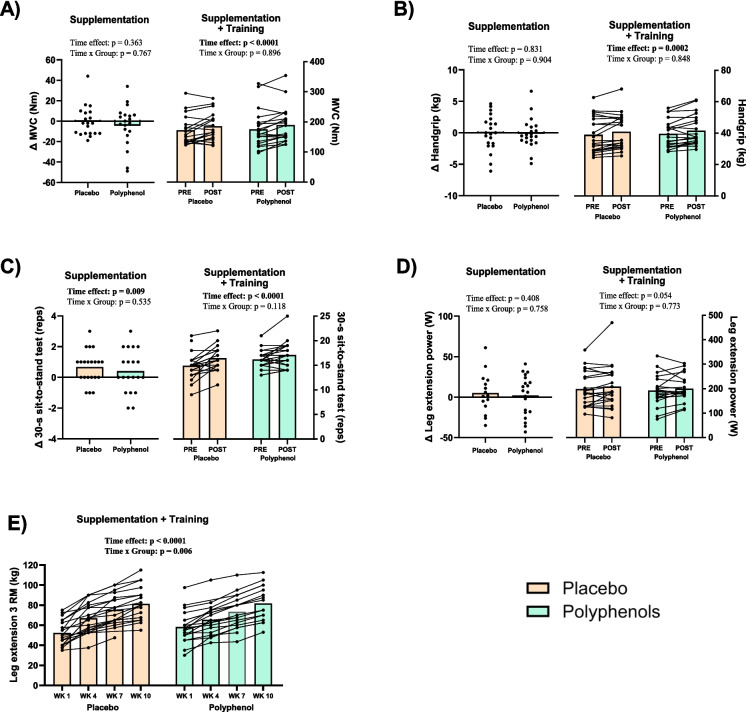
Muscle and functional strength. Strength measurements in **A**–**D** are shown as the individual responses (delta values) to 1 month of supplementation with placebo or polyphenol (left part of each set of graphs, supplement effect) and as the individual responses 3 months of training + supplementation, placebo or polyphenol (right part of each set of graphs). **A** Maximal voluntary contraction (MVC) strength (Nm), determined by isometric dynamometer. **B** Handgrip strength (kg) determined by dynamometer. **C** Thirty-second sit to stand test reps. **D** Leg extension power (W) determined by Power Rig. **E** Leg extension 3 repetition max (3RM), measured at weeks 1, 4, 7, and 10 during the training intervention. Polyphenol supplementation is shown with green bars and placebo with beige bars. Data presented as individual values and mean (bars). For delta graphs, *p*-values reflect statistics done on total values, though graphs are expressed as delta values (change from screening visit to PRE visit, before training intervention). *p*-value < 0.05 is considered significant and indicated in bold

The training intervention increased MVC by 9.6 ± 13.1% in the placebo group and by 10.3 ± 12.1% in the polyphenol group (time effect: *p* < 0.0001), with no differences between groups (time × group effect: *p* = 0.896) (Fig. 1A). The intervention did not result in any changes in RFD in any time interval in either group (time effect: *p* = 0.598, data not shown). Handgrip strength increased by 1.9 ± 3.3 kg in the placebo group and by 2.1 ± 3.1 kg in the polyphenol group (time effect: *p* = 0.0002), with no differences between groups (time × group effect: *p* = 0.848) following the training intervention (Fig. 1B). The loading phase resulted in a 1 ± 1 rep increase in STS test reps in both the placebo and polyphenol group (time effect: *p* = 0.009), with no differences between groups (time × group effect: *p* = 0.535) (Fig. 1C). No other strength measurements were changed following the loading phase. Following the training intervention, STS test reps increased by 2 ± 2 reps in the placebo group, and by 1 ± 2 reps in the polyphenol group (time effect: *p* < 0.0001), with no differences between groups (time × group effect: *p* = 0.118) (Fig. 1C). LEP tended to increase by 5 ± 15% in the placebo group and by 8 ± 17% in the polyphenol group (time effect: *p* = 0.054, with no differences between groups (time × group effect: *p* = 0.773) (Fig. 1D). From week 1 to week 10, leg extension 3RM increased by 29 ± 15 kg in the placebo group and by 22 ± 7 kg in the polyphenol group (time effect: *p* < 0.0001), with a significant difference between groups (time × group effect: *p* = 0.006) (Fig. 1E). Post hoc multiple comparisons revealed no significant difference between groups at any specific timepoint.

### Cycling performance and gas exchange

**Fig. 2:**
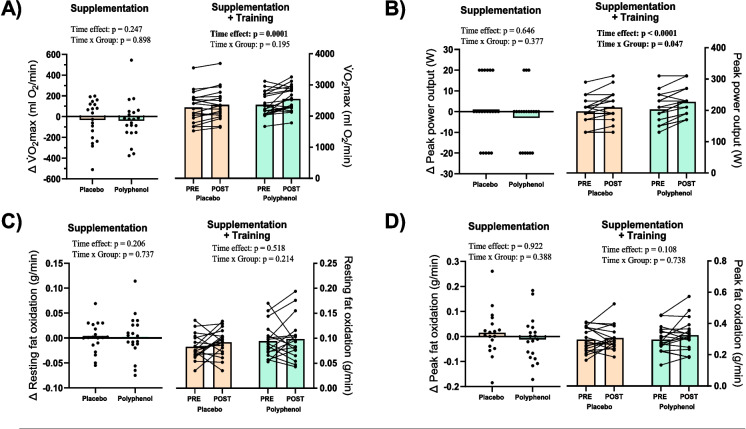
Cardiorespiratory fitness and fat oxidation. Cardiorespiratory fitness and fat oxidation measurements are shown as the individual responses (delta values) to 1 month of supplementation with placebo or polyphenol (left part of each set of graphs, supplement effect) and as the individual responses to three month of training + supplementation, placebo or polyphenol (right part of each set of graphs). **A** Maximal oxygen uptake (ml O_2_/min) determined by ramp test. **B** Peak power output achieved during VO2max ramp test. **C** Fat oxidation (g/min) determined at rest during maximal fat oxidation test. **D** Peak fat oxidation (g/min) determined during maximal fat oxidation test. Polyphenol supplementation are shown with green bars and placebo with beige bars. Data presented as individual values and mean (bars). For delta graphs, *p*-values reflect statistics done on total values, though graphs are expressed as delta values (change from screening visit to PRE visit, before training intervention). *p*-value < 0.05 considered significant and indicated in bold

Following the training intervention, V̇O_2_max increased by 125 ± 131 ml O_2_/min in the placebo group and by 130 ± 204 ml O_2_/min in the polyphenol group (time effect: *p* = 0.0001), with no differences between groups (time × group effect: *p* = 0.195) (Fig. 2A). Peak power output in the V̇O_2_max test increased by 12 ± 18 W in the placebo group and by 24 ± 17 W in the polyphenol group (time effect: *p* < 0.0001) after the training intervention, with a significant difference between groups (time × group effect: *p* = 0.047) (Fig. 2B). The loading phase did not affect any parameters of the V̇O_2_max test.

**Fig. 3:**
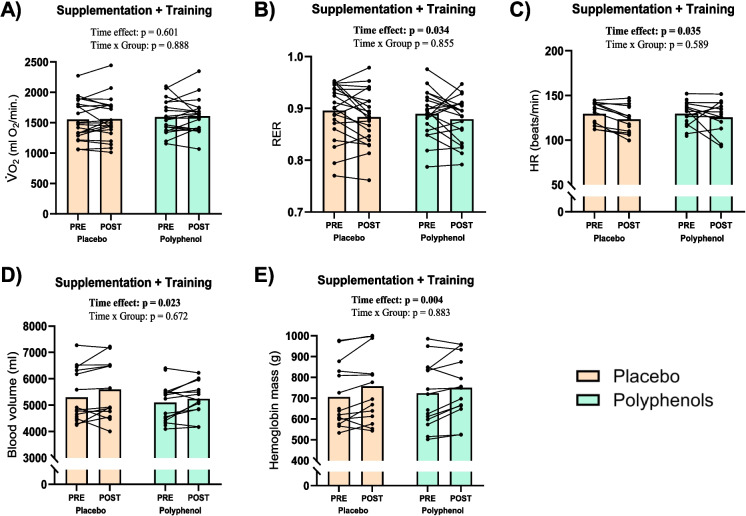
Acute physiological exercise response + hematological changes. Gas exchange, heart rate, and hematological measurements before (PRE) and after 12 weeks of training (POST) with continuous polyphenol or placebo supplementation. **A** Average oxygen uptake (ml O_2_/min) from minute 15 to minute 45 during 45-min moderate intensity cycling test. **B** Average respiratory exchange ratio from minute 15 to minute 45 during 45-min moderate intensity cycling test. **C** Average heart rate (beats/min) from minute 15 to minute 45 during 45-min moderate intensity cycling test. **D** Total blood volume (ml) determined by carbon monoxide rebreathing, measured at rest. **E** Total hemoglobin mass (g) determined by carbon monoxide rebreathing, measured at rest. Polyphenol supplementation are shown with green bars and placebo with beige bars. Data presented as individual values and mean (bars). *p*-value < 0.05 considered significant and indicated in bold

There were no significant differences in V̇O_2_ during the 45-min cycling test before and after the training intervention and no differences between the two groups (Fig. 3A). Following the training intervention, the average relative percentage of V̇O_2_max during the cycling test decreased from 69 to 66% (time effect: *p* = 0.018), with no difference between groups (time × group effect: *p* = 0.197). For both groups, there was a main effect of time on RER which decreased from 0.90 ± 0.05 to 0.88 ± 0.05 in the placebo group and from 0.89 ± 0.04 to 0.88 ± 0.04 in the polyphenol group in the POST test compared to the PRE test (time effect: *p* = 0.034), with no differences between groups (time × group effect: *p* = 0.855) (Fig. 3B). A similar main effect of time was observed for average heart rate, which decreased from 129 ± 12 bpm to 123 ± 15 bpm in the placebo group and from 130 ± 15 bpm to 125 ± 17 bpm in the polyphenol group (time effect: *p* = 0.035), with no differences between groups (time × group effect: *p* = 0.589) (Fig. 3C).

No statistically significant differences were observed in resting fat oxidation or peak fat oxidation, neither in total values (Fig. 2C, D) nor when normalized to lean mass (not shown), for either group following the supplement loading phase or the training intervention.

### Blood volume and hemoglobin mass

There was a main effect of time on blood volume (time effect: *p* = 0.023), which increased by 291 ± 345 mL in the placebo group and by 139 ± 312 mL in the polyphenol group, with no differences between groups (time × group effect: *p* = 0.672) (Fig. 3D) following the training intervention. Total hemoglobin mass increased significantly in both groups as a result of the training intervention, by 7.4 ± 6.6% in the placebo group and by 3.6 ± 6.2% in the polyphenol group (time effect: *p* = 0.004) with no differences between groups (time × group effect: *p* = 0.883) (Fig. 3E).

### Plasma metabolites

Following the loading phase, plasma total cholesterol decreased by 0.4 ± 0.5 mmol/L in the placebo group and by 0.6 ± 0.5 mmol/L in the polyphenol group (time effect: *p* = 0.008), with no differences between groups (time × group effect: *p* = 0.287) (Table 3). Multiple comparisons revealed a significant difference only in the polyphenol group (*p* < 0.05). Plasma HDL decreased by 0.1 ± 0.2 mmol/L in the placebo group and by 0.2 ± 0.2 mmol/L in the polyphenol group (time effect: *p* = 0.003), with a tendency for differences between groups (time × group effect: *p* = 0.070) (Table 3), following the loading phase. Multiple comparisons revealed a significant decrease only in the polyphenol group (*p* = 0.006). Following the loading phase, plasma FFA increased by 134 ± 257 µmol/L in the placebo group and by 92 ± 299 µmol/L in the polyphenol group (time effect: *p* = 0.015), with no differences between groups (time × group effect: *p* = 0.633) (Table 3). No other metabolites were altered following the loading phase. No changes were observed for glucose, lactate, insulin, ketone, glycerol, triglyceride, or cortisol levels following the training intervention (Table 3).

**Table 3:** Plasma metabolites

	Placebo (*n* = 21)			Polyphenol (*n* = 20)			*p*-value SCR to PRE		*p*-value PRE to POST	
	SCR	PRE	POST	SCR	PRE	POST	Time	Time × group	Time	Time × group
Total cholesterol (mmol/L)	6.2 ± 1.5	5.8 ± 1.3	5.2 ± 1.2	6.1 ± 1.0	5.5 ± 1.1	5.2 ± 1.0	**0.008**	0.287	**0.010**	0.439
LDL cholesterol (mmol/L)	3.6 ± 0.9	3.3 ± 0.8	3.0 ± 0.8	3.5 ± 0.7	3.3 ± 0.8	2.9 ± 0.9	0.061	0.982	**0.042**	0.652
HDL cholesterol (mmol/L)	1.7 ± 0.7	1.6 ± 0.7	1.6 ± 0.6	1.8 ± 0.4	1.6 ± 0.4	1.7 ± 0.4	**0.003**	0.070	0.644	0.066
Free fatty acids (µmol/L)	398 ± 176	532 ± 219	579 ± 201	472 ± 236	564 ± 287	635 ± 285	**0.015**	0.633	0.112	0.743
Triglycerides (mmol/L)	1.4 ± 1.0	1.3 ± 0.7	1.2 ± 0.5	1.3 ± 0.5	1.3 ± 0.6	1.2 ± 0.5	0.314	0.378	0.574	0.756
Glycerol (µmol/L)	82 ± 26	95 ± 37	98 ± 43	92 ± 39	94 ± 43	99 ± 44	0.216	0.317	0.454	0.831
Ketones (mmol/L)	0.06 ± 0.06	0.07 ± 0.06	0.08 ± 0.07	0.11 ± 0.12	0.10 ± 0.12	0.12 ± 0.15	0.873	0.589	0.411	0.470
Glucose (mmol/L)	5.4 ± 0.7	5.3 ± 0.5	5.4 ± 0.5	5.4 ± 0.8	5.4 ± 0.7	5.2 ± 0.5	0.633	0.347	0.582	0.110
Insulin (pmol/L)	53 ± 37	64 ± 52	55 ± 34	46 ± 22	55 ± 36	48 ± 22	0.067	0.858	0.259	0.380
Lactate (pmol/L)	1.1 ± 0.4	0.9 ± 0.2	0.8 ± 0.2	1.1 ± 0.5	1.0 ± 0.4	1.0 ± 0.4	0.176	0.171	0.588	0.465
Cortisol (nmol/L)	361 ± 98	333 ± 100	360 ± 117	368 ± 114	346 ± 86	358 ± 65	0.181	0.856	0.380	0.723

Plasma metabolites measured at rest at the screening visit (SCR), after one month of polyphenol or placebo supplementation (PRE) and after 12 weeks of training with continuous supplementation (POST). Data were analyzed using a two-way ANOVA. *p*-value < 0.05 was considered significant and is indicated in bold. Data are presented as means ± SD

During the moderate intensity exercise test and subsequent recovery period, there was a significant main effect of time (duration of the test) on all plasma metabolites, meaning that all plasma metabolites fluctuated significantly, with no significant differences between groups in the PRE tests (after supplement loading, before training). There was a significant main effect of the training intervention leading to lower plasma lactate levels during exercise and recovery in both the placebo group (*p* < 0.0001) and the polyphenol group (*p* < 0.0001) (Table S3). Cortisol levels were significantly lower during exercise and recovery in the placebo group (*p* < 0.05, main effect of intervention) following the training intervention, while no significant difference was observed in the polyphenol group (Table S3). Glycerol levels were significantly lower in the polyphenol group during exercise and recovery (*p* < 0.0001, main effect of intervention) following the training intervention, while no significant difference was observed in the placebo group (Table S3). No other plasma metabolites were significantly altered during exercise and recovery following the training intervention (Table S3).

No plasma inflammatory markers were significantly altered following the loading phase, though we observed a tendency for lower IL-18 (time effect: *p* = 0.079) and IL-6 (time effect: *p* = 0.058), with no differences between groups (Table 4). No plasma inflammatory markers were altered at rest following the training intervention (Table 4).

**Table 4:** Plasma inflammatory markers

	Placebo (*n* = 21)			Polyphenol (*n* = 20)			*p*-value SCR to PRE		*p*-value PRE to POST	
	SCR	PRE	POST	SCR	PRE	POST	Time	Time × group	Time	Time × group
IL-10 (pg/ml)	3.8 ± 4.4	3.9 ± 4.4	3.9 ± 4.7	3.4 ± 4.2	3.5 ± 4.6	4.2 ± 5.5	0.568	0.851	0.174	0.153
IL-18 (pg/ml)	1703 ± 877	1612 ± 760	1550 ± 765	1612 ± 802	1559 ± 818	1530 ± 823	0.079	0.804	0.377	0.704
IL-6 (pg/ml)	3.0 ± 1.9	2.9 ± 1.7	2.7 ± 1.5	2.6 ± 1.5	2.3 ± 1.1	2.3 ± 1.1	0.058	0.423	0.480	0.126
IL-8 (pg/ml)	11.2 ± 5.4	10.8 ± 4.6	11.3 ± 5.4	11.2 ± 4.5	10.6 ± 4.2	10.4 ± 4.4	0.189	0.830	0.638	0.276
IFN-γ (pg/ml)	158 ± 208	165 ± 185	166 ± 217	129 ± 222	118 ± 201	138 ± 233	0.866	0.368	0.416	0.364
MCP-1 (pg/ml)	313 ± 174	341 ± 203	319 ± 192	345 ± 180	328 ± 173	334 ± 173	0.937	0.135	0.899	0.384
TNF-α (pg/ml)	2.2 ± 1.1	2.2 ± 1.0	2.3 ± 1.2	2.0 ± 1.2	1.8 ± 1.0	2.0 ± 1.2	0.165	0.241	0.143	0.337
IL-1β (pg/ml)	0.7 ± 0.6	0.6 ± 0.5	0.5 ± 0.6	0.5 ± 0.3	0.4 ± 0.2	0.5 ± 0.2	0.123	0.886	0.752	0.096
IL-4 (pg/ml)	1.2 ± 2.1	1.2 ± 2.3	1.2 ± 2.0	1.1 ± 1.9	1.0 ± 1.3	1.0 ± 2.3	0.485	0.289	0.653	0.573
CRP (mg/L)	1.9 ± 1.7	1.6 ± 1.5	1.4 ± 1.9	1.2 ± 0.9	1.1 ± 1.3	1.0 ± 0.8	0.470	0.728	0.535	0.826

Plasma inflammatory markers measured at rest at the screening visit (SCR), after one month of polyphenol or placebo supplementation (PRE) and after 12 weeks of training with continuous supplementation (POST). Data were analyzed using a two-way ANOVA. *p*-value < 0.05 was considered significant and is indicated in bold. Data are presented as means ± SD

**Fig. 4:**
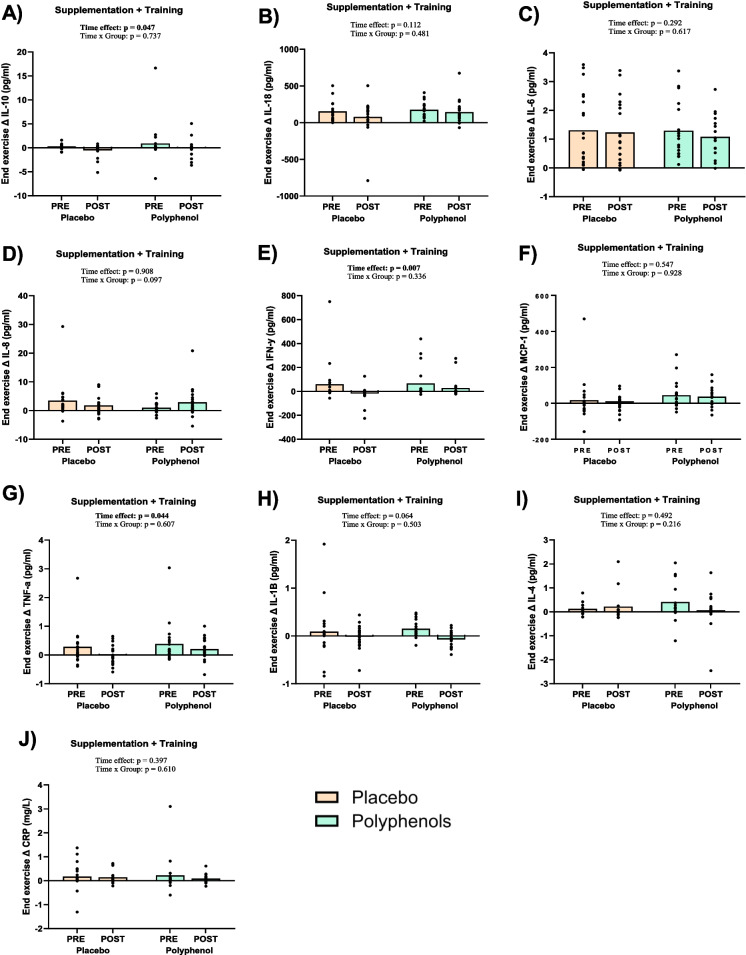
Plasma inflammatory markers. Plasma inflammatory markers before (PRE), and after 12 weeks of training (POST) with continuous polyphenol or placebo supplementation, measured at rest and at the end of 45 min of moderate intensity cycling, expressed as change from baseline (rest) to the end of exercise (Δ value). **A** Interleukin 10 (pg/ml). **B** Interleukin 18 (pg/ml). **C** Interleukin 6 (pg/ml). **D** Interleukin 8 (pg/ml). **E** Interferon gamma (pg/ml). **F** Monocyte chemoattractant protein-1 (pg/ml). **G** Tumor necrosis factor alpha (pg/ml). **H** Interleukin 1β (pg/ml). **I** Interleukin 4 (pg/ml). **J** C-reactive protein (mg/L). Polyphenol supplementation is shown with green bars and placebo with beige bars. Data presented as individual values and mean (bars). *p*-value < 0.05 considered significant and indicated in bold

When analyzing the change in inflammatory markers from baseline (rest) to the end of exercise (minute 45), there was a significant main effect of the intervention on IL-10 levels (time effect: *p* = 0.047) resulting in decreased Δ values in response to exercise, with no difference between groups (time × group effect: *p* = 0.737) (Fig. 4A). The change in IFN- γ in response to exercise was significantly decreased following the training intervention (time effect: *p* = 0.007, main effect) with no difference between groups (time × group effect: *p* = 0.336) (Fig. 4E). The exercise-induced increase in TNF- α was significantly lowered following the training intervention (time effect: *p* = 0.044) with no difference between groups (time × group effect: *p* = 0.607) (Fig. 4G). IL-1B displayed a trend towards decreased response to exercise (time effect: *p* = 0.064) with no difference between groups (time × group effect: *p* = 0.503) (Fig. 4H). No other markers displayed a significantly altered response to exercise (Fig. 4) (Table S2).

### Gene expression

No significant differences were observed in the relative expression of the selected inflammatory-related genes MCP-1, TLR4, and NF-KB between experimental conditions (Fig. S7). Gene expression levels remained consistent across all samples, suggesting that the intervention did not influence the transcription of these targets. Ct values were normalized to the reference gene ACTB, and statistical analysis confirmed the absence of significant changes (*p* > 0.05).

## Discussion

Aging is associated with gradual declines in muscle mass, strength, aerobic capacity, metabolic efficiency, and increased low-grade inflammation, all of which contribute to frailty, cardiovascular disease, and metabolic dysfunction. This study investigated the effects of RT combined with a small amount of HIIT, with or without polyphenol supplementation, on body composition, metabolic and cardiovascular adaptations, muscle fiber characteristics, and inflammatory responses in aging individuals. Our findings demonstrate that structured exercise training elicits significant improvements in several indicators of muscle mass and strength, with concomitant increases in indicators of aerobic capacity and substrate utilization efficiency, demonstrating promising effects on overall health and physical capacity associated with healthy aging and longevity. Notably, training appeared to blunt the response to exercise of some inflammatory markers, indicating a more regulated inflammatory profile that may contribute to improved health and muscle function. Polyphenol supplementation had minimal beneficial effects, though some health benefits cannot be ruled out.

### Muscle mass and strength

Training resulted in significant increases in whole-body lean mass, thigh lean mass, vastus lateralis CSA, and type 2 muscle fiber CSA, indicating hypertrophy of both whole muscle and individual fast-twitch fibers. The ability of resistance-type training to increase muscle mass in aging and elderly individuals is well established [24–34], and here we add to the findings by showing robust increases in several markers of muscle mass, from whole-body and leg-specific down to the cellular level, following RT combined with HIIT. We observed selective type II muscle fiber hypertrophy, which is in line with most [27, 28, 30, 34–38] but not all [26, 39, 40] RT studies in aging and elderly individuals. Given that age-related loss of muscle mass predominantly results from the atrophy of type II muscle fibers [41], preserving these fibers is crucial for maintaining strength and power in aging populations. Only a few studies have examined the effects of combined RT and HIIT in elderly individuals, showing either a modest (0.5 ± 2.2%) [42] or no increase [43] in lean mass, with only a trend towards increased muscle fiber CSA [43]. Here we show that the combination of RT and HIIT can elicit increases in muscle mass comparable to RT alone, also in aging individuals, indicating no interference effect of HIIT, contrary to previous speculation [44]. Polyphenols alone did not affect body composition and did not result in any additional gains in lean mass when combined with training. Few studies have reported increases in various muscle mass indices after polyphenol supplementation alone [45, 46], or in combination with resistance training [47], while one study reported no changes [48]. However, all studies included sarcopenic individuals, whereas the present study included healthy individuals. In addition, there are major differences between the composition and dosage of polyphenol supplements used across studies, making comparisons increasingly difficult. In the present study, we focused on a berry-based supplement of about 700 mg of polyphenols per day, as described above (supplementary data S8), for 16 weeks, whereas the studies showing an effect in sarcopenic individuals employ various types of catechins with doses varying between 70 and 540 mg of polyphenols and treatment periods varying between 4 and 24 weeks. As such, the discrepancy in findings between previous studies and the present study is likely not related to dosage or treatment period, but rather to the type of polyphenols and patient groups included.

Similarly, training significantly enhanced all measures of muscle strength, which is in line with the very well-documented efficacy of resistance-type training in enhancing muscle strength in elderly individuals [49], often to a greater extent than muscle hypertrophy. Here we demonstrate the potent effect of this type of intervention on absolute muscle contractile strength (MVC, handgrip strength, and 3RM), functional muscle strength (sit-to-stand test), and a strong tendency towards increased muscle power (Power rig), all key factors in mitigating the age-related decline in physical capacity. Again, the combination with HIIT did not seem to have detrimental effects on strength gains, which is in line with existing literature on aging individuals [43, 44]. Polyphenols did not affect any measures of strength or physical function. Some studies in sarcopenic individuals have reported improvements in tests of physical function or strength following polyphenol supplementation alone [46, 50] or in combination with exercise [45, 48], while one study found no differences following supplementation alone [51]. Collectively, our results do not support the ability of polyphenols to enhance muscle mass or strength in elderly individuals, though a potential benefit in frail elderly individuals or of other types of polyphenols cannot be ruled out.

### Aerobic capacity

The training intervention resulted in an average 6% increase in V̇O_2_max, which is in line with previous studies in healthy older adults showing approximately an 8% increase in V̇O_2_max following either 12 [43] or 16 [42] weeks of RT combined with HIIT. The potential of HIIT alone to increase V̇O_2_max in elderly individuals is well established [52, 53], with increases ranging from 5% and up to 25% after various types of HIIT interventions. Although the increase in V̇O₂max was modest compared to previous reports, it was achieved with only a single weekly HIIT session comprising 7–9 min of high-intensity work, substantially less than in typical HIIT or concurrent training protocols. Given that V̇O₂max is a key predictor of all-cause mortality and cardiovascular health, independent of traditional risk factors such as blood pressure, cholesterol, and smoking [54, 55], even modest gains are clinically meaningful. Further evidence of improved aerobic capacity included significantly lower heart rate and plasma lactate concentrations observed during the 45-min cycling test at a fixed absolute workload. Together with the increase in V̇O₂max, this suggests enhanced cardiorespiratory efficiency and highlights the effectiveness of the training intervention in improving aerobic function in older adults. Improvements in V̇O₂max after 12 weeks of training are likely driven by peripheral adaptations, such as increased capillarization and mitochondrial density, whereas central (cardiac) adaptations have previously been shown to require a longer duration [56]. However, a novel finding of this study was that 12 weeks of RT combined with minimal HIIT increased both total blood volume and hemoglobin mass, which contributes to increased cardiorespiratory fitness. Haematological adaptations have previously been shown to be the main driver of increased V̇O₂max after short-term endurance training protocols [57], and this is the first study to show increased blood volume and hemoglobin mass following a primarily RT-based protocol in aging individuals. To our knowledge, only two previous studies have shown increased blood volume following RT, one in untrained men [58] and one in well-trained rowers [59]. The mechanisms remain somewhat elusive, but it is likely related to increased erythropoietin secretion and release of vasopressin and aldosterone following an increased oxygen demand [60]. Collectively, these findings demonstrate that RT combined with minimal HIIT can significantly enhance aerobic capacity in older adults, likely mediated by hematological adaptations.

Polyphenol supplementation did not enhance any indices of aerobic capacity, and to our knowledge, no studies have assessed the effects of polyphenol supplementation on aerobic capacity in older adults. In younger, active, or trained populations, polyphenol supplementation has not been shown to improve V̇O₂max [61–65], yet some studies have reported improvements in measurements of endurance performance [61, 65–67]. While the current study did not include a dedicated performance test, we did observe a significant group × time interaction in peak power output during the V̇O₂max test, suggesting greater training-induced gains in the polyphenol group compared to placebo. As such, a performance-enhancing effect of polyphenol supplementation cannot be entirely ruled out; however, the lack of consistent improvements across other measures of aerobic capacity or muscular strength weakens this interpretation. Finally, as noted above, comparisons across studies are difficult due to differences in compounds and treatment duration. Studies demonstrating ergogenic effects have employed dosages ranging from 120 to 750 mg of polyphenols over treatment periods of 7 to 28 days. In contrast, only a potential effect was observed in the present study, suggesting that the lack of a clear effect may be more attributable to differences in the patient population or the specific polyphenol formulation used. Taken together, our findings provide limited support for a meaningful effect of polyphenol supplementation on aerobic capacity or endurance performance, either alone or in conjunction with exercise training.

### Metabolic adaptations

The supplement loading phase resulted in significantly lower total cholesterol levels (main effect of time) likely driven by the polyphenol group as multiple comparisons revealed a significant reduction only in the polyphenol group. Though the literature is somewhat divergent, some clinical studies have reported significant decreases in both total and LDL cholesterol following polyphenol supplementation [68, 69]. Despite this decline in cholesterol levels during the loading phase, the training intervention led to further significant reductions in both total and LDL cholesterol levels, without significant reductions in body weight or fat mass. This is particularly relevant in older adults, as elevated cholesterol and LDL levels are key risk factors for cardiovascular disease mortality, the incidence of which increases with age. Surprisingly, HDL cholesterol was significantly lower following the loading phase, likely driven by the polyphenol group as multiple comparisons revealed a significant reduction only in the polyphenol group and the group × time difference came close to significance (*p* = 0.07). Following the loading phase, HDL levels decreased in 13 of 20 participants and increased in 3 in the polyphenol group. In the placebo group, levels decreased in 8 and increased in 7 of 21 participants. To our knowledge, current evidence indicates that polyphenol supplementation either increases HDL or has no effect. Thus, the observed reduction in HDL is more plausibly attributed to a change in habitual behavior or dietary patterns among the participants during the loading phase. This is supported by the fact that we also observed significantly higher levels of plasma FFA following the loading phase, in both groups, despite participants being asked to maintain habitual diet and physical activity levels. However, we did not detect any changes in body composition, weight, fitness level, fat oxidation, or any other plasma metabolites following the loading phase.

Following the training intervention, participants exhibited significantly lower RER and plasma lactate concentrations during submaximal cycling at the same workload, indicating a shift toward greater fat oxidation and enhanced metabolic efficiency. Similarly, plasma cortisol levels were reduced in both groups, though it only reached statistical significance in the placebo group. This attenuated cortisol response may reflect enhanced exercise tolerance and substrate utilization, reducing the exercise-induced stress response and hormonal support required to meet energy demands. Though this remains speculative, peak fat oxidation showed a trend toward improvement after the training intervention, further indicating enhanced fat metabolism. Studies investigating the effects of RT on fat oxidation are scarce, but some indicate of a beneficial effect. One study reported increased 24-h fat oxidation in older women following 16 weeks of RT [70], while another found a reduced RER during sleep in young adults after 6 months of RT [71]. Although the training intervention in the present study was primarily RT-based, it incorporated HIIT as well, which has been shown to enhance peak fat oxidation to the same extent as moderate-intensity endurance training [72].

In summary, the present findings highlight the capacity of RT and HIIT, even in the absence of weight loss, to improve markers of metabolic health and substrate utilization in aging adults. The potential effects of polyphenols appeared modest; however, the reduction in cholesterol levels suggests a favorable impact on lipid metabolism and potential cardiovascular benefits. As previously noted, the relative exercise intensity during the post-intervention moderate-intensity cycling test was slightly lower (66% vs. 69% of V̇O₂max) due to increased V̇O₂max, despite the same absolute workload. This reduction in relative intensity may partially explain some of the observed reductions in metabolic stress markers. Nevertheless, the reduction was minor and the breadth and consistency of the changes across RER, lactate, cortisol, and trends in fat oxidation suggest genuine physiological adaptations beyond a mere reduction in relative effort. Together, these results emphasize the value of targeted training interventions in promoting metabolic resilience with advancing age.

### Inflammation

Neither polyphenol supplementation, training, nor their combination led to significant changes in circulating inflammatory markers. This aligns with most previous studies reporting no effect of RT on systemic inflammation in healthy older adults [73–77]. Some studies do show reductions in circulatory inflammatory markers following RT, but these effects occur alongside reductions in fat mass, which may confound the interpretation of these findings. Systemic inflammation has been shown to correlate with both total and visceral fat mass [78, 79], and reductions in fat mass have been associated with decreased circulating inflammatory markers [80], raising the possibility that fat loss rather than training itself may drive the observed effects. One study found that RT led to lower CRP and TNF-α [81]; however, these effects occurred with concomitant reductions in central fat mass and fat percentage. Two other studies found similar reductions in inflammatory markers but did not include data on body composition changes [82, 83]. Nevertheless, one study reported significant reductions in CRP in older adults after 32 weeks of resistance training [84], despite no changes in BMI or waist circumference. Notably, no effects were observed at 16 weeks, suggesting that reductions in systemic inflammation may require longer-term RT interventions than are typically employed. Moreover, we observed no changes in muscle mRNA levels of the selected inflammatory gene expression markers following RT, further indicating no effect of the training or supplementation intervention on basal muscle inflammatory status. To our knowledge, no clinical studies have investigated the effects of polyphenol supplementation, either alone or in combination with exercise training, on circulatory inflammatory markers in healthy older adults. Some studies have demonstrated that polyphenol supplementation can reduce circulating inflammatory markers in individuals with type 2 diabetes [85, 86] and patients undergoing hemodialysis [87], suggesting that their anti-inflammatory effects may be more pronounced in populations with elevated baseline inflammation.

While no effects on basal systemic or muscle inflammation were observed, aging has also been associated with an exaggerated inflammatory response to acute exercise [11, 12]. Interestingly, when looking at the delta values of plasma inflammatory markers during exercise (end of exercise values relative to resting values), the training intervention appeared to attenuate the exercise-induced inflammatory response, suggesting improved inflammatory regulation with training. This altered inflammatory response to exercise was evident for IL-10, IFN-γ, and TNF-α, with a strong trend observed for IL-1β and a similar, though non-significant, pattern for IL-18, IL-6, and MCP-1. Though this interpretation is somewhat speculative, 12 weeks of RT has previously been shown to attenuate the exercise-induced inflammatory response in muscle in older adults [10]. Similarly, lifelong exercisers display a blunted inflammatory response to exercise compared to sedentary age-matched individuals, resembling that of younger trained adults, suggesting that regular exercise can modulate age-related inflammatory reactivity. This may be of particular importance in aging individuals, as the age-related increased inflammatory response may impair training adaptations [10, 11, 88], whereas attenuating inflammation pharmacologically has been shown to enhance adaptive responses in older adults [89, 90]. Polyphenol supplementation did not seem to affect the inflammatory response to exercise, and to our knowledge no other studies have investigated this in older adults. However, some previous studies in young adults have shown reduced inflammatory markers in the days following eccentric-induced muscle damage when ingesting polyphenol supplements [91, 92], while some did not report any differences [93].

In summary, while neither polyphenol supplementation nor RT altered basal inflammatory status, the observed attenuation of the exercise-induced inflammatory response suggests that training may enhance inflammatory regulation in older adults. This adaptive modulation could be functionally relevant for improving recovery and optimizing training outcomes in aging populations. The role of polyphenols in this context remains unclear, but our results do not support a meaningful anti-inflammatory role, warranting further investigation.

## Conclusion and perspectives

This study shows that 12 weeks of RT combined with minimal HIIT can significantly improve muscle mass, strength, aerobic capacity, and metabolic health in older adults. These adaptations occurred without weight loss and were supported by indications of a shift toward greater fat oxidation during exercise and, notably, increases in blood volume and hemoglobin mass, a novel finding in this population that may contribute to improved oxygen delivery and aerobic function. The training intervention did not alter basal systemic or muscle inflammation but appeared to attenuate the acute inflammatory response to exercise, indicating improved inflammatory regulation with potential relevance for recovery and long-term adaptation in aging individuals. Polyphenol supplementation had minimal effects and did not meaningfully enhance training adaptations, though modest benefits in cholesterol levels suggest potential relevance for cardiovascular health. Together, these results highlight the broad benefits of targeted RT-based exercise interventions for healthy aging, beyond just strength and muscle mass, and emphasize the potential of well-designed exercise interventions to mitigate the age-related physiological decline, even in the absence of pharmacological treatment. Future research should investigate longer interventions, different training modalities, and populations with greater baseline inflammation or frailty to clarify the role of polyphenols and optimize intervention strategies for healthy aging.

## Supplementary Information

Below is the link to the electronic supplementary material.

Supplementary file1 (PDF 383 KB)

Supplementary file2 (PDF 367 KB)

## Data Availability

Data will be made available upon request to the corresponding author.
