# Supplementary material for: Effects of resistance-based training and polyphenol supplementation on physical function, metabolism, and inflammation in aging individuals
Source: GeroScience. 2025 Aug 19;48(2):2945–68. doi: 10.1007/s11357-025-01839-8 (PMC12972354; doi:10.1007/s11357-025-01839-8)
Supplement: Supplementary file 1 — Supplementary file1 (PDF 383 KB) [file 11357_2025_1839_MOESM1_ESM.pdf]

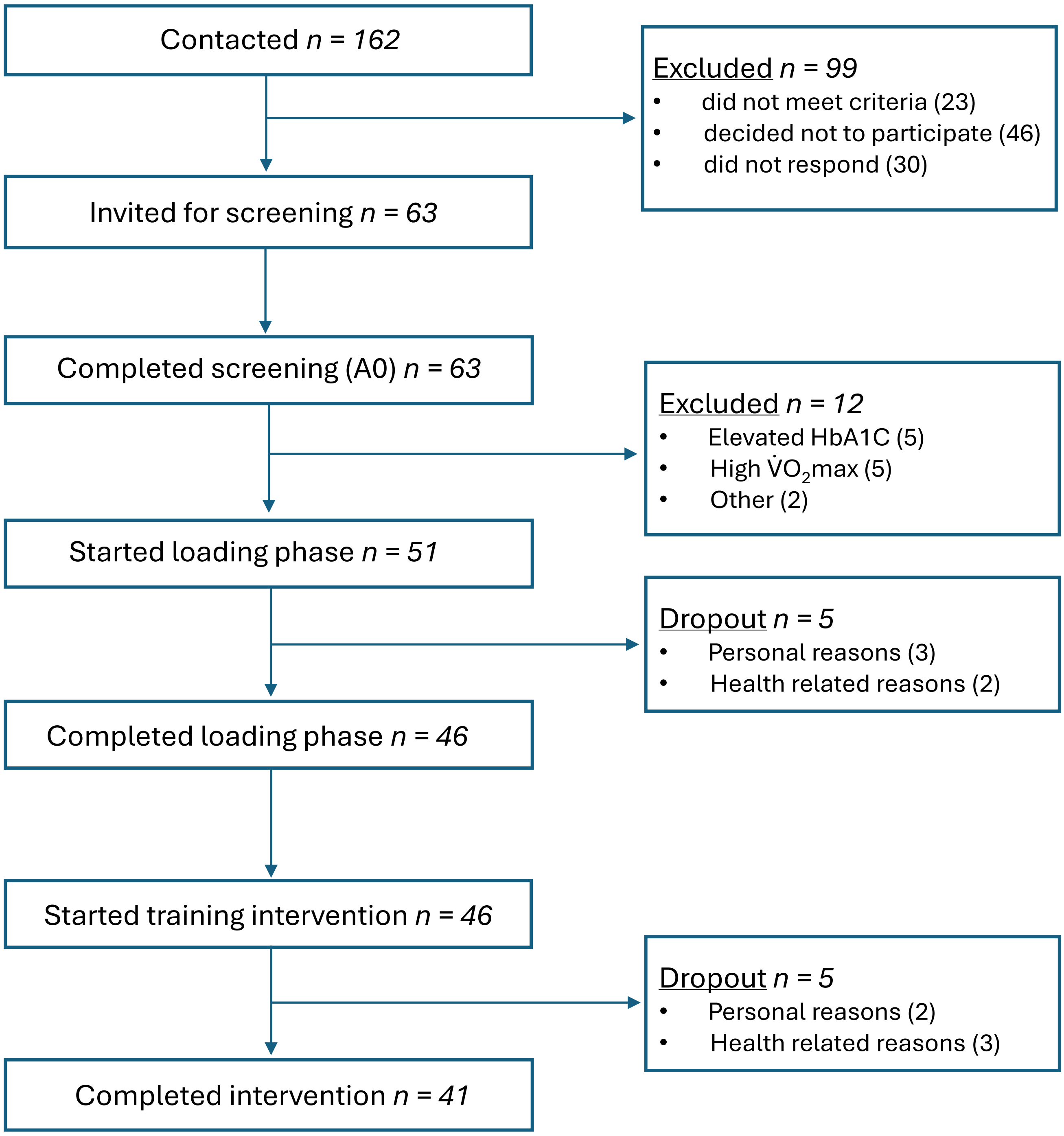

Supplementary figure S1. Consort diagram of number of subjects contacted, screened, excluded and included in the study. Eligibility criteria described in methods section.

Placebo			Exercise			Recovery		Polyphenol	Exercise			Recovery	
		-15	15	30	45	60	105	-15	15	30	45	60	105
IL-10 (pg/ml)	PRE	3.9±4.2	3.6±4.1	3.5±3.8	3.8±4.3	4.6±5.1	4.1±3.7	3.4±4.5	3.7±4.8	4.0±5.4	4.0±7.1	3.6±4.3	3.3±3.8
	POST	3.9±4.6	3.8±4.5	4.1±4.8	3.3±3.8	3.6±4.1	3.0±3.0	4.4±5.3	4.6±5.3	5.3±6.6	4.4±5.2	4.0±4.6	4.2±4.8
IL-18 (pg/ml)	PRE	1612±742	1753±792	1774±789	1751±799	1696±803	1566±759	1559±798	1728±882	1760±932	1729±883	1591±812	1489±745
	POST	1550±765	1678±786	1688±796	1717±764	1550±736	1517±709	1530±823	1689±887	1716±886	1700±886	1552±816	1383±699
IL-1β (pg/ml)	PRE	0.64±0.51	0.67±0.68	0.61±0.54	0.68±0.75	0.63±0.91	0.59±0.54	0.38±0.20	0.40±0.21	0.45±0.22	0.53±0.22	0.48±0.31	0.52±0.27
	POST	0.52±0.56	0.59±0.72	0.58±0.53	0.54±0.63	0.69±1.07	0.64±0.68	0.50±0.20	0.58±0.51	0.52±0.25	0.47±0.25	0.43±0.18	0.47±0.22
IL-4 (pg/ml)	PRE	1.2±2.2	1.0±1.5	1.6±2.7	1.7±2.5	1.6±2.4	1.3±2.1	1.0±1.3	1.1±2.0	1.0±1.8	1.3±1.9	0.9±2.1	0.9±1.7
	POST	1.2±2.0	1.3±2.1	1.4±2.5	1.3±2.4	1.4±2.3	1.2±1.8	1.0±2.2	1.0±1.8	1.2±2.2	1.0±1.8	1.0±1.8	1.0±1.7
IL-6 (pg/ml)	PRE	2.9±1.6	3.4±1.8	3.5±1.8	4.2±2.1	3.9±2.2	4.1±2.0	2.4±1.1	2.7±1.2	2.9±1.2	3.5±1.3	3.9±1.8	4.4±2.0
	POST	2.7±1.5	3.0±1.7	3.1±1.7	3.7±1.7	3.6±1.8	4.2±2.2	2.3±1.1	2.7±1.3	2.8±1.2	3.4±1.2	3.7±1.8	3.7±2.0
IL-8 (pg/ml)	PRE	10.8±4.5	10.6±3.7	12.4±5.1	14.2±9.1	13.2±8.4	12.0±4.2	10.6±4.1	9.6±3.5	11.5±4.6	11.5±4.3	10.0±3.0	12.3±5.2
	POST	11.2±5.2	12.3±4.5	12.7±6.2	13.1±5.5	12.0±6.2	11.0±4.3	10.4±4.3	11.9±6.9	12.8±6.1	12.8±6.1	12.4±5.7	11.6±4.4
IFN-γ (pg/ml)	PRE	165±180	157±184	184±204	225±295	207±240	171±205	118±196	136±236	142±254	187±322	128±226	116±209
	POST	166±212	177±217	176±221	142±186	151±186	171±223	138±227	166±298	172±312	167±303	164±303	158±274
MCP-1 (pg/ml)	PRE	341±198	327±185	316±172	343±188	323±191	327±200	328±169	347±158	355±171	366±168	361±166	369±204
	POST	319±187	335±187	324±184	336±199	322±192	330±199	334±169	338±174	361±164	384±175	368±190	384±196
TNF-α (pg/ml)	PRE	2.2±1.0	2.1±0.9	2.3±1.1	2.5±1.3	2.2±1.0	2.2±0.9	1.8±1.0	2.0±1.1	2.2±1.3	2.2±1.3	2.0±1.1	1.8±1.0
	POST	2.3±1.1	2.4±1.1	2.3±1.1	2.3±1.1	2.3±1.1	2.2±1.0	2.0±1.1	2.2±1.3	2.3±1.3	2.2±1.3	2.2±1.2	1.9±1.1
CRP (mg/L)	PRE	1.9±1.8	2.0±2.0	2.1±2.1	2.0±2.1	2.0±1.9	1.8±1.9	1.1±1.3	1.4±1.4	1.4±1.4	1.4±1.4	1.2±1.3	1.1±1.1
	POST	1.4±1.9	1.6±2.0	1.6±2.0	1.6±2.0	1.5±1.8	1.4±1.8	1.0±0.8	1.2±0.9	1.2±0.9	1.2±0.9	1.0±0.8	0.9±0.7

Supplementary table S2. Plasma inflammatory markers at rest (-15), during 45 minutes of moderate intensity exercise (15, 30 45) and 1 hour of recovery (60, 105) before (PRE) and after (POST) 12 weeks of training. Data were analyzed using a two-way ANOVA. P-value < 0.05 was considered significant. Data are presented as means ± SD.

		Placebo	Exercise			Recovery				Polyphenol	Exercise			Recovery			
		-15	15	30	45	60	75	90	105	-15	15	30	45	60	75	90	105
Glucose (mmol/L)	PRE	5.3±0.5	5.4±0.6	5.5±0.7	5.7±0.8	5.8±0.8	5.5±0.7	5.3±0.6	5.2±0.5	5.4 ± 0.8	5.4±0.6	5.4±0.6	5.7±1.0	5.7±0.6	5.5±0.8	5.2±0.6	5.1±0.7
	POST	5.4±0.5	5.5±0.7	5.7±0.9	5.8±1.0	5.8±1.0	5.5±0.9	5.2±0.8	5.2±0.7	5.2 ± 0.5	5.3±0.6	5.3±0.6	5.4±0.7	5.6±0.8	5.3±0.8	5.1±0.7	5.0±0.6
Insulin (pmol/L)	PRE	64±52	52±33	47±33	82±70	113±70	81±67	60±55	55±56	55±36	41±20	34±14	47±45	101±57	76±45	45±23	35±15
	POST	55±34	41±21	47±30	69±45	108±77	75±61	48±37	53±42	48±22	34±16	29±12	58±40	94±48	67±36	43±29	42±24
Lactate (pmol/L)	PRE	0.9±0.2	2.6±1.4	2.8±1.8	2.9±1.8	1.8±1.0	1.4±0.7	1.1±0.5	1.0±0.4	1.1±0.4	3.4±1.2	3.2±1.4	3.5±2.3	2.2±1.5	1.5±0.4	1.4±0.7	1.4±0.7
	POST	**0.8±0.2***	**1.9±1.2***	**2.2±1.5***	**2.2±1.6***	**1.4±0.9***	**1.1±0.5***	**1.0±0.3***	**0.9±0.3***	**1.0±0.4***	**2.3±1.0***	**2.3±1.2***	**2.4±1.6***	**1.6±0.9***	**1.3±0.5***	**1.1±0.4***	**1.5±1.9***
Cortisol (nmol/L)	PRE	333±100	325±130	384±159	463±172	463±193	422±185	375±156	400±107	346±86	329±103	389±145	433±172	457±191	425±189	365±174	323±154
	POST	**360±117***	**303±104***	**334±134***	**358±156***	**345±158***	**298±130***	**264±111***	**306±108***	358±65	321±90	351±137	389±172	400±180	349±175	316±161	315±119
Ketones (mmol/L)	PRE	0.06±0.08	0.05±0.04	0.06±0.05	0.1±0.08	0.23±0.17	0.24±0.2	0.19±0.16	0.15±0.13	0.10±0.13	0.07±0.04	0.08±0.07	0.12±0.09	0.27±0.2	0.3±0.25	0.26±0.24	0.19±0.19
	POST	0.08±0.08	0.05±0.03	0.06±0.05	0.1±0.06	0.29±0.15	0.31±0.2	0.25±0.15	0.18±0.11	0.12±0.14	0.07±0.07	0.09±0.08	0.12±0.11	0.31±0.27	0.35±0.3	0.29±0.32	0.20±0.25
FFA (µmol/L)	PRE	532±219	475±225	608±321	853±417	1133±535	837±318	616±216	543±192	564±287	514±210	641±320	811±435	1385±603	1072±455	759±280	612±166
	POST	579±201	484±177	637±268	787±292	1288±437	957±357	702±230	574±112	635±285	488±185	615±243	797±315	1310±455	930±255	627±146	533±89
Glycerol (µmol/L)	PRE	95±37	141±56	219±92	271±113	183±72	117±49	87±31	82±29	94±43	164±85	247±123	325±146	242±94	155±61	105±33	88±26
	POST	98±43	149±57	236±93	302±108	211±99	126±55	90±32	74±21	**99±44***	**151±68***	**223±90***	**280±108***	**206±69***	**126±52***	**82±28***	**74±25***
TG (mmol/L)	PRE	1.3±0.7	1.4±0.8	1.5±0.8	1.5±0.7	1.4±0.7	1.2±0.7	1.2±0.7	1.0±0.4	1.3±0.6	1.5±0.6	1.6±0.6	1.7±0.6	1.4±0.4	1.3±0.5	1.2±0.5	1.2±0.5
	POST	1.3±0.5	1.4±0.4	1.5±0.4	1.5±0.4	1.3±0.4	1.2±0.3	1.1±0.3	1.1±0.3	1.2±0.5	1.4±0.5	1.4±0.5	1.5±0.5	1.3±0.5	1.1±0.4	1.1±0.4	1.1±0.4
Cholesterol (mmol/L)	PRE	5.8±1.3	6.2±1.6	6.4±1.5	6.2±1.4	5.7±1.3	5.4±1.3	5.4±1.3	5.3±1.3	5.5±1.1	6.2±1.2	6.2±1.2	6.3±1.1	5.7±1.1	5.4±1.0	5.3±1.0	5.3±1.0
	POST	5.2±1.2	5.8±1.3	5.8±1.3	5.9±1.4	5.4±1.2	4.9±1.6	5.0±1.2	4.9±1.3	5.2±1.0	5.8±1.2	5.8±1.3	5.8±1.3	5.4±1.2	5.2±1.2	5.0±1.1	5.0±1.1
LDL (mmol/L)	PRE	3.3±0.8	3.6±1.0	3.7±1.0	3.6±0.9	3.3±0.8	3.1±0.8	3.2±0.8	3.1±0.8	3.3±0.8	3.6±0.9	3.6±0.9	3.7±0.9	3.4±0.9	3.2±0.8	3.1±0.8	3.1±0.8
	POST	3.1±0.8	3.4±0.9	3.5±0.9	3.5±0.9	3.2±0.8	3.0±1.0	3.0±0.8	3.0±0.8	2.9±0.9	3.4±1.0	3.4±1.1	3.4±1.1	3.2±1.0	3.0±1.0	3.0±0.9	2.0±0.9
HDL (mmol/L)	PRE	1.6±0.7	1.8±0.8	1.8±0.7	1.8±0.7	1.6±0.5	1.5±0.5	1.5±0.6	1.6±0.6	1.6±0.4	1.8±0.4	1.8±0.4	1.8±0.4	1.7±0.4	1.6±0.4	1.6±0.4	1.5±0.4
	POST	1.6±0.6	1.7±0.7	1.7±0.7	1.8±0.7	1.6±0.5	1.5±0.5	1.5±0.6	1.5±0.5	1.7±0.4	1.8±0.4	1.8±0.4	1.8±0.4	1.7±0.4	1.6±0.4	1.6±0.4	1.6±0.4

Supplementary table S3. Plasma metabolites at rest (-15), during 45 minutes of moderate intensity exercise (15, 30, 45) and 1 hour of recovery (60, 75, 90, 105) before (PRE) and after (POST) 12 weeks of training. Data were analyzed using a two-way ANOVA. P-value < 0.05 was considered significant, and is indicated in bold. Data are presented as means ± SD. In the case of a main effect, all timepoint values are marked as follows: * = main effect of training intervention.

Interval number	1	2	3	4	5	6	7	8	9
**Placebo**									
**Week 1**	87±8	91±5	98±6	99±4	100±7	105±22	107±16	-	-
**Week 2**	88±8	93±6	102±5	103±6	102±7	115±18	108±12	-	-
**Week 3**	90±7	94±7	100±5	101±4	101±5	117±17	114±12	-	-
**Week 4**	90±7	95±6	102±4	102±6	100±7	118±16	115±18	-	-
**Week 5**	89±7	91±8	104±5	106±6	105±6	106±8	120±16	117±12	-
**Week 6**	90±6	93±4	106±5	105±5	106±5	104±11	120±16	109±12	-
**Week 7**	91±5	95±6	106±4	106±3	106±4	105±4	121±19	115±11	-
**Week 8**	89±6	92±4	106±4	106±4	107±4	106±4	121±19	112±15	-
**Week 9**	90±5	92±3	108±5	109±5	108±5	109±7	116±15	119±12	112±11
**Week 10**	89±4	90±7	109±5	110±5	109±5	109±5	119±19	116±12	113±14
**Week 11**	90±3	93±3	111±4	111±3	109±3	109±5	113±15	110±10	113±15
**Week 12**	90±6	94±5	109±6	109±6	109±6	109±4	115±17	114±11	116±10
**Polyphenol**									
**Week 1**	86±7	92±6	102±7	101±5	100±5	108±11	103±12	-	-
**Week 2**	89±11	94±7	102±7	103±6	100±5	108±14	104±13	-	-
**Week 3**	89±9	92±5	100±6	100±8	98±7	115±19	105±16	-	-
**Week 4**	89±9	91±5	101±6	102±4	102±5	112±12	114±13	-	-
**Week 5**	91±11	92±6	103±7	105±5	104±4	103±6	114±13	112±17	-
**Week 6**	86±10	92±5	103±6	105±6	104±5	105±6	116±16	109±26	-
**Week 7**	89±10	91±6	104±7	106±7	104±5	104±5	119±18	115±21	-
**Week 8**	91±8	92±7	105±6	106±4	107±5	106±4	118±14	116±16	-
**Week 9**	92±6	93±7	108±8	107±8	109±6	110±9	113±10	114±16	108±9
**Week 10**	90±6	91±6	108±7	109±6	108±6	108±6	121±17	115±19	109±19
**Week 11**	89±6	89±7	109±5	109±5	109±5	109±5	120±13	119±21	113±15
**Week 12**	89±8	90±7	109±5	109±5	110±7	107±5	120±14	121±17	113±16

Supplementary table S4. Average power output recorded for each 60 second cycling interval during 12 weeks of HIIT (1 weekly session), expressed as % of the power output that elicited VO_2max during a VO_2max test. Data are presented as means ± SD.

Interval number	1	2	3	4	5	6	7	8	9
**Placebo**									
**Week 1**	81±8	84±8	87±9	90±5	91±6	96±6	95±4	-	-
**Week 2**	79±9	84±6	89±6	91±6	91±6	95±3	97±3	-	-
**Week 3**	81±6	85±6	88±7	90±5	91±6	96±3	97±2	-	-
**Week 4**	80±10	85±7	88±6	89±6	90±6	95±4	96±4	-	-
**Week 5**	79±7	83±6	86±6	89±6	90±6	91±7	94±5	96±3	-
**Week 6**	79±8	84±6	88±7	90±6	90±7	90±6	94±4	96±3	-
**Week 7**	81±7	83±8	87±6	90±5	90±6	91±6	95±4	95±4	-
**Week 8**	80±6	82±6	87±6	89±6	90±7	90±6	95±3	95±4	-
**Week 9**	81±6	83±8	87±5	90±5	91±5	91±5	94±4	96±3	96±3
**Week 10**	78±10	81±7	87±7	89±6	91±7	93±7	95±5	97±3	96±4
**Week 11**	82±6	83±7	88±7	91±6	91±7	92±7	94±6	95±5	95±5
**Week 12**	80±6	84±7	90±6	92±5	93±4	94±5	96±2	96±4	97±3
**Polyphenol**									
**Week 1**	81±7	85±6	90±6	92±7	93±6	96±3	97±2	-	-
**Week 2**	82±5	85±5	89±5	92±4	93±4	96±3	96±4	-	-
**Week 3**	81±10	86±6	89±6	91±6	93±5	97±2	97±2	-	-
**Week 4**	79±11	85±6	89±5	90±6	91±6	95±4	97±2	-	-
**Week 5**	80±8	83±7	87±7	89±7	91±6	91±6	96±4	97±2	-
**Week 6**	77±10	83±9	88±5	90±6	91±6	93±5	97±2	97±3	-
**Week 7**	81±5	82±6	88±5	89±6	92±6	91±6	96±2	97±2	-
**Week 8**	82±6	84±5	87±7	91±5	92±4	93±4	96±3	97±2	-
**Week 9**	78±7	83±7	88±5	90±6	91±6	94±5	95±5	96±3	97±2
**Week 10**	79±5	81±5	87±4	88±4	90±4	91±4	95±4	95±4	96±4
**Week 11**	77±4	81±5	87±5	90±4	92±4	92±4	95±4	97±2	96±4
**Week 12**	80±8	81±6	85±6	89±6	90±6	90±5	93±4	95±5	95±5

Supplementary table S5. Peak heart rate recorded for each 60 second cycling interval during 12 weeks of HIIT (1 weekly session), expressed as % maximal heart rate recorded. Data are presented as means ± SD.

Gene	Primer	Sequence (5' to 3')
NF-Kβ	Forward	CGCCGCTTAGGAGGGAGA
	Reverse	CCATTCTGAAGCCGGGTGG
TLR4	Forward	CGTGGAGACTTGGCCCTAAA
	Reverse	AAGCAATGGAATCGGGGTGA
MCP-1	Forward	CCCAAGCAGAAGTGGGTTCA
	Reverse	GTGTCTGGGGAAAGCTAGGG
Actin-β	Forward	CTGGAACGGTGAAGGTGACA
	Reverse	AAGGGACTTCCTGTAACAATGCA

Supplementary table S6. qPCR primer sequences for Nuclear factor kappa-light-chain-enhancer of activated B cells (NF-K β), Toll-like receptor 4 (TLR4), monocyte chemoattractant protein 1 (MCP-1) and Actin-β.

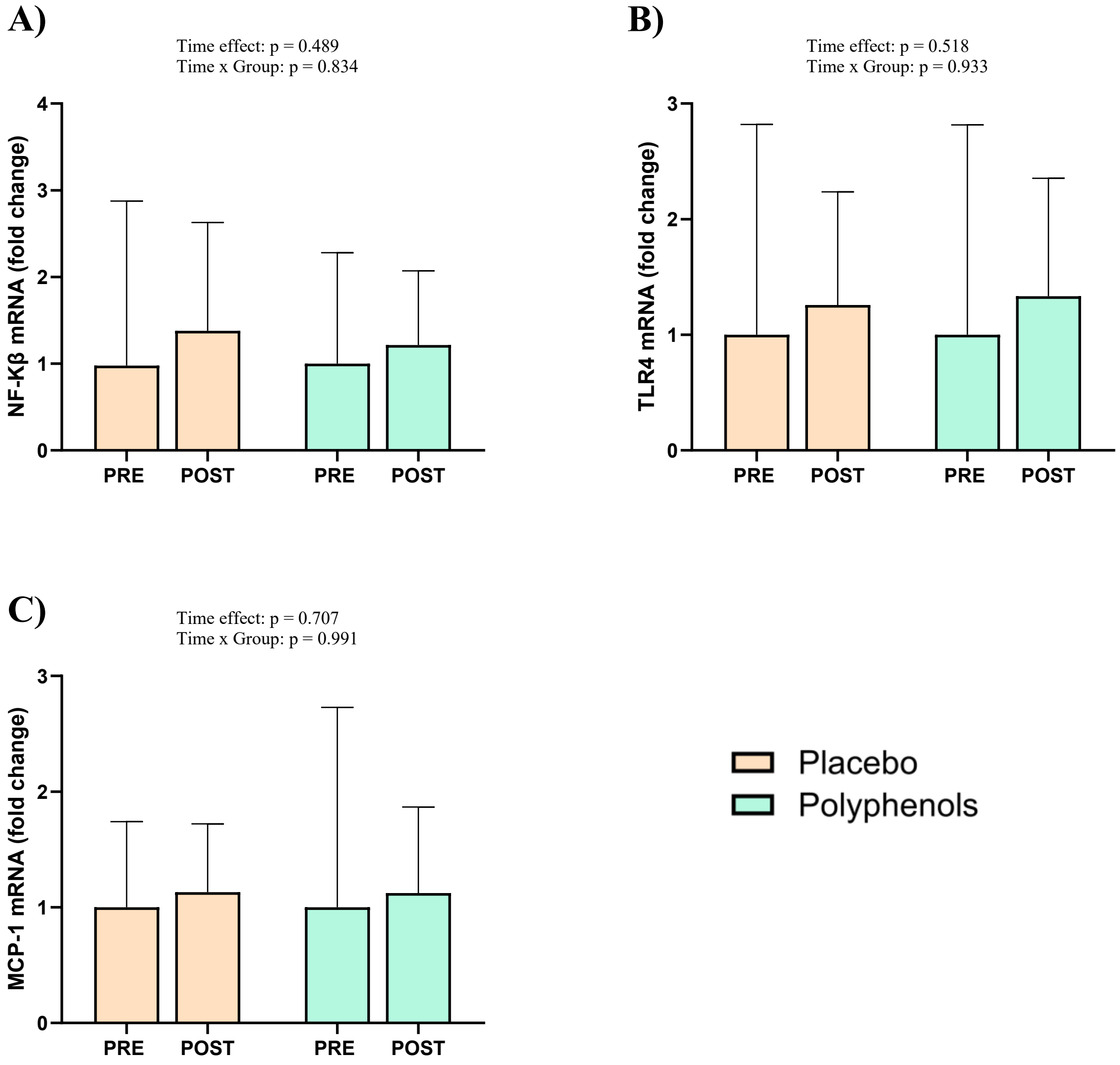

Supplementary figure S7. Muscle mRNA levels determined by qPCR and expressed as fold change, after 30 days of polyphenol (green bars) or placebo (beige bars) supplementation (PRE), and after 12 weeks of training (POST) with continuous supplementation. **A)** Nuclear Factor Kappa B. **B)** Toll-like Receptor 4. **C)** Monocyte chemoattractant protein-1. Data presented as mean ± SD. P-value < 0.05 considered significant and indicated in bold.
