# Supplementary material for: Effects of resistance-based training and polyphenol supplementation on physical function, metabolism, and inflammation in aging individuals
Source: GeroScience. 2025 Aug 19;48(2):2945–68. doi: 10.1007/s11357-025-01839-8 (PMC12972354; doi:10.1007/s11357-025-01839-8)
Supplement: Supplementary file 2 — Supplementary file2 (PDF 367 KB) [file 11357_2025_1839_MOESM2_ESM.pdf]

Analysis of MitoActive and Placebo

Analyses:

- Total polyphenol determination
- Monomeric anthocyanin determination
- LC-MS analysis
  - Ribetrils
  - Targeted analysis of polyphenols in MitoActive
  - Targeted analysis of *Beta vulgaris* compounds in the placebo
  - FlavoFinder analysis of flavonoids and phenolic acids
  - Molecular network analysis
  - UpSet-analysis (“Venn diagram”)

Total polyphenol determination

Total polyphenol content was determined using the Folin-Ciocalteu assay, using gallic acid as the reference compound.

MitoActive 0.98±0.01% (gallic acid equivalents)

Placebo 0.09±0.004% (gallic acid equivalents)

Monomeric anthocyanin determination

Monomeric anthocyanin content was determined using the pH differential method, using cyanidin 3-glucoside as reference compound.

MitoActive 0.005 ± 0.003% (cyanidin 3-glucoside equivalents)

Placebo none (result of analysis: -0.043 ± 0.001%)

LC-MS analysis

<u>Ribetrils</u>

The polyphenols in MitoAcive (red- and blackcurrants) are a mix of mainly flavonoids (including anthocyanins, flavonols, flavanols, etc) and phenolic acids, as well as ribetrils.

Ribetril A and its four analogues ribetril B-E were all detected by LC-MS and verified by MS/MS fragmentation, through detection of each of their characteristic phenolic acid fragments. No ribetrils were present in the placebo.

Name	Formula	$[M-H]^-$	Phenolic acid fragment m/z (ESI-)	Phenolic acid
Ribetril A	C20H23NO10	436.1244	179.034	Caffeic acid
Ribetril B	C21H25NO10	450.1397	193.05	Ferulic acid
Ribetril C	C20H23NO9	420.1295	163.039	p-coumaric acid
Ribetril D	C19H23NO10	424.1244	167.035	Vanillic acid
Ribetril E	C18H21NO9	394.1138	137.024	4-hydroxy benzoic acid

<u>Targeted analysis of polyphenols in MitoActive</u>

List of selected polyphenols commonly found in red- and blackcurrants detected in MitoActive. Hits have been filtered and only include those with integrated areas above 10000.

Polyphenol	ESI- detection	ESI+ detection	MS2 match	Confidence level*
3-Caffeoylquinic acid	+	-	-	3
3-p-Coumaroylquinic acid	+	-	-	3
4-Caffeoylquinic acid	+	-	-	3
4-Hydroxybenzoic acid 4-O-glucoside	+	-	ESI-	2
4-p-Coumaroylquinic acid	+	-	-	3
5-Caffeoylquinic acid	+	-	-	3
Caffeic acid 4-O-glucoside	+	-	ESI-	2
cyanidin 3-O-glucoside	+	+	ESI-/ESI+	2
Cyanidin 3-O-rutinoside	+	+	ESI-/ESI+	2
Delphinidin 3-O-(6''-p-coumaroylglucoside)	-	+	-	3
Delphinidin 3-O-glucoside	+	+	ESI+	2
Delphinidin 3-O-rutinoside	+	+	ESI-	2
Ferulic acid 4-O-glucoside	+	-	ESI-	2
Isorhamnetin 3-O-rutinoside	+	-	-	3
Kaempferol 3-O-glucoside	+	+	ESI-/ESI+	2
Kaempferol 3-O-rutinoside	+	+	ESI-/ESI+	2
Myricetin 3-O-glucoside	+	+	ESI-/ESI+	2
Myricetin 3-O-rutinoside	+	+	ESI-/ESI+	2
p-Coumaric acid 4-O-glucoside	+	-	ESI-	2
Peonidin 3-O-rutinoside	-	+	-	3
Petunidin 3-O-rutinoside	+	-	-	3
Prodelphinidin B3-B4	-	+	-	3
Quercetin 3-O-glucoside	+	+	ESI-/ESI+	2
Quercetin 3-O-rutinoside	-	+	ESI-/ESI+	2

* Based on Reisdorph *et al.*, 2018

Confidence levels are based on Reisdorph *et al*., 2018. Level 3 hits are MS1-only matches, i.e. correct m/z values (within 10 ppm), but without MS2 confirmation (mainly caused by lack of MS2 scans due to low intensity). Level 2 hits are confirmed with a combination of GNPS library matches, SIRIUS compound class prediction, and/or manual or automated MS2 matching. Automated MS matching of flavonoids and

phenolic acids were done using an in-house developed script that searches either raw or processed fragmentation data for characteristic flavonoids and phenolic acid aglycone fragments.

An additional search using a target list with ~220 *Ribes sp.* secondary metabolites from the reaxys database, gave 31 and 43 hits in positive and negative ionization respectively.

<u>Targeted analysis of *Beta vulgaris* compounds in the placebo</u>

Searching for polyphenols from beet root (= the roots of *Beta vulgaris*) in databases such as reaxys, only gives a very limited number of compounds. The polyphenolic compounds were comprised mainly of various ferulic acid derivatives, and only a limited number of flavonoids, including the flavanone dihydroisorhamnetin and the hydroxyisoflavone betavulgarin.

Due to the low levels of polyphenols in the placebo, compounds other than polyphenolics were included in the target list. Compounds such as carbohydrates or amino acids are not included. The target list was generated using the reaxys database and included compounds isolated from beet root (=the roots of *Beta vulgaris*).

**Compound**	**ESI- detection**	**ESI+ detection**	**MS2 match**	**Confidence level***
17-Decarboxy-betanin	+	-	+	2
1-Feruloyl-β-D-glucopyranoside	-	-	-	-
Betalamic acid	-	+	+	2
Betanin	+	+	+	2
Betavulgarin	-	-	-	-
Neobetanin	+	+	-	3
Xanthine	-	-	-	-

* Based on Reisdorph *et al.*, 2018

Only a very limited number of compounds could be confidently detected in the placebo, none of which were phenolic compounds. Signature compounds such as betanin was detected.

Flavonoid analysis using FlavoFinder

FlavoFinder is a tool developed at Asiros Nordic that uses fragmentation data to quickly and automatically identify tentative flavonoids and phenolic acids in LC-MS data by searching for fragments matching the flavonoid or phenolic acid aglycones.

As expected, as larger number of both flavonoids and phenolic acids are found in MitoActive compared to the placebo.

**ESI-**	MitoActive	Placebo
Flavonoids	26	10
Phenolic acids	44	8

**ESI+**	MitoActive	Placebo
Flavonoids	23	9
Phenolic acids	13	7

Molecular network analysis

A feature based molecular network was generated using the processed LC-MS data set, consisting of SPE treated MitoActive, two SPE treated placebo samples (one using the standard SPE washing volume and one using a reduce volume), and instrument blanks. Processing was done using MZMine 3. Molecular formulae and compound class predictions (used during compound dereplication) were generated using SIRIUS 5.8.2, FlavoFinder results were included for further confirmation.

Selected clusters of tentatively identified compounds from the network analysis. Red color indicates compounds found in MitoActive, and green indicates compounds found in the placebo.

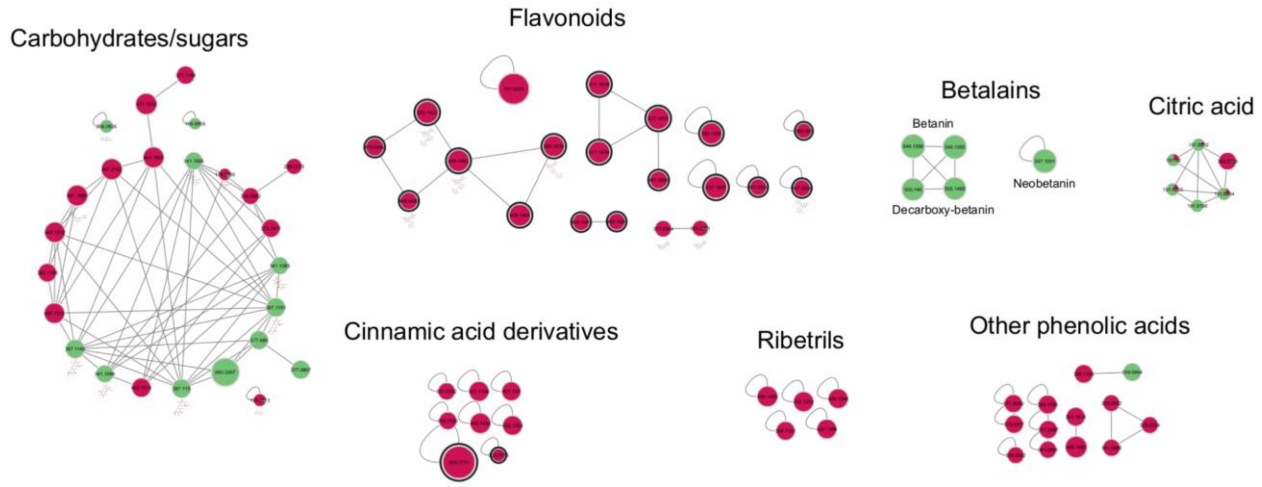

UpSet analysis

An UpSet plot is similar to a Venn diagram, but is able to better display a higher number of sets.

An UpSet plots was made for all features present after processing of the LC-MS dataset. The processing was done using MZMine 3, and any features found in the instrument blanks were removed before generating the plot.

A total of 650 features were found in the samples, of which 415 were exclusive to MitoActive and 134 to the placebo. 101 features were shared.

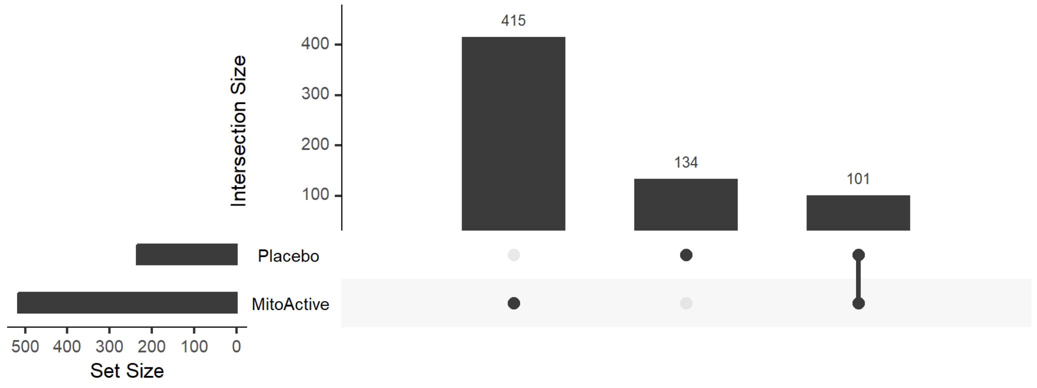

A similar analysis of only flavonoids (as identified by FlavoFinder), showed that out of 20 tentative flavonoids, only one was present in the placebo. Upon further analysis, this compound turned out to be betain, i.e. a false positive from FlavoFinder.

Experimental procedure

Total polyphenol determination, FC assay

Total polyphenol content was determined using the Folin–Ciocalteu (F-C) assay using gallic acid as the reference compound.

<u>Preparation of samples, standards and reagents:</u>

Samples were prepared in duplicate by accurately weighing ~400mg in a 50 mL volumetric flask, filling with MQ water and mixing the solution by inverting the flask 4-5 times.

A **standard** dilution row of gallic acid was made by first dissolving 25 mg of gallic acid in a 25 mL volumetric flask and filling partially with MQ water. The solution was sonicated for 10 minutes and the remaining volume was filled and the solution was mixed by inverting the flask producing a stock solution with a concentration of 1000 µg/mL. Two dilution rows were produced from the stock. The first concentration used in the dilution row (200 µmg/mL) was made by diluting 200 µL of the stock solution with 800 µL MQ water, followed by repeated 2x dilutions with MQ water until a final concentration of 6.25 µg/mL.

The **F-C reagent** was prepared by adding 2.5 mL of a 10% F-C reagent solution in a volumetric flask, filling up with MQ water, and mixing by repeated inversions of the flask.

A 700 mM **sodium carbonate** solution was prepared by dissolving 74.2 g of Na_2CO_3 in 1 L of MQ water. The solution was stirred until everything was dissolved.

<u>Procedure</u>

Each sample solution was measured in triplicates, giving a total of six measurements per sample.

In a 96-deep well plate, 100 µL sample or standard was mixed with 200 µL F-C reagent. The mixtures were allowed to mix, before 800 µL of sodium carbonate solution was added to start the reaction. The reaction was allowed to incubate for 1 hour in the dark before transferring 200 µL to a flat bottom 96-well plate and reading absorbance values at 760nm. The absorbance values were used to calculate percent total polyphenol content as gallic acid equivalents.

Monomeric anthocyanin determination, pH differential assay

Total monomeric anthocyanin content was determined using the pH differential method, using cyanidin 3-glucoside as the reference compound.

Preparation of samples, and reagents:

A KCl buffer, pH 1, was prepared by first mixing 1.86 g KCl with ~950 mL MQ water. The solution was adjusted to pH 1 with 6 M HCl, and the remaining volume was filled with MQ water.

A sodium acetate buffer, pH 4.5, was prepared by first mixing 54.43 g sodium acetate trihydrate with ~950 mL MQ water. The solution was adjusted to pH 4.5 with 6 M HCl, and the remaining volume was filled with MQ water.

Samples were prepared in duplicates for each pH.

Solutions of ~15 mg/mL were prepared by accurately weighing ~750 mg of sample in a 50 mL volumetric flask and filling with either KCl or sodium acetate buffer. The solutions were mixed by repeated inversions and allowed to incubate for 20 min in the dark.

Absorbance was measured at 520 nm and 720 nm and monomeric anthocyanin content was determined using the formula;

$$\text{Anthocyanin content [\%]} = \Delta A \cdot \frac{MW \cdot DF \cdot V}{\varepsilon \cdot l} \cdot 100\%$$

Where,

$$\Delta A = \left(\frac{A520nm - A720nm}{m[pH\ 1]}\right)[\text{pH } 1] - \left(\frac{A520nm - A720nm}{m[pH\ 4.5]}\right)[\text{pH } 4.5]$$

MW = molecular weight of cyanidin 3-glucoside (=449.2 g/mol)

DF = dilution factor (=1)

V = volume used to dissolve sample (=50mL)

ε = molar absorption coefficient for cyanidin 3-glucoside (=26900 $L \cdot mol^{-1} \cdot cm^{-1}$)

l = path length of 96-well plate (=1 cm, path length is corrected to 1 cm using blanks in the instrument software)

Sample preparation for LC-MS analysis

Before LC-MS analysis, the samples were subjected to SPE clean up using Biotage Isolute C18(EC) columns (100 mg, 1 mL).

- SPE columns were first conditioned by passing 3 CVs of MeOH through each column, followed by 3 CVs of MQ water.
- Samples (200 µL) were loaded and 3 CVs MQ water is added to wash the column. For the placebo, an additional sample was made using only 1 mL for the washing step, to prevent early elution of betalain pigments.
- Samples were eluted by adding 3 CVs of MeOH, and the eluate were collected.
- The eluates were dried under at stream of nitrogen at 30-35 °C.

- The dried samples were resuspended in 400 mL of 1:1 MeOH:MQ water, and transferred (200-300 µL) to HPLC vial with inserts for LC-MS analysis.

LC-MS analysis

LC-MS analysis was carried out on an Agilent 1290 LC systems connected to an Agilent 6545 QTOF mass spectrometer. A volume of 5 µL was injected onto a Waters Acquity UPLC HSS T3 C18 (100x2.1mm, 100Å, 1.8 µm), using water in line A and acetonitrile in line B, both buffered with 20 mM formic acid. The gradient started at 5% B increasing to 40% over 10 min, followed by an increase to 100%B in 0.1 min, washing at 100%B for two minutes, and return to 5%B in 0.1 min for re-equilibration for 2 minutes.

Data processing

Description of molecular network parameters:

The molecular network was created with the Feature-Based Molecular Networking (FBMN) workflow (Nothias L-F, Petras D, Schmid R et al. Nature Methods 17, 905–908 (2020)) on GNPS (https://gnps.ucsd.edu, Wang M et al. Nat. Biotech. 2016). The mass spectrometry data were first processed with MZMine3 and the results were exported to GNPS for FBMN analysis. The data was filtered by removing all MS/MS fragment ions within +/- 17 Da of the precursor m/z. MS/MS spectra were window filtered by choosing only the top 6 fragment ions in the +/- 50 Da window throughout the spectrum. The precursor ion mass tolerance was set to 0.02 Da and the MS/MS fragment ion tolerance to 0.02 Da. A molecular network was then created where edges were filtered to have a cosine score above 0.65 and more than 6 matched peaks. Further, edges between two nodes were kept in the network if and only if each of the nodes appeared in each others respective top 10 most similar nodes. Finally, the maximum size of a molecular family was set to 100, and the lowest scoring edges were removed from molecular families until the molecular family size was below this threshold. The spectra in the network were then searched against GNPS spectral libraries (Cite Wang M, et al. Nature Biotech. 2016 and Horai, H. et al. J. Mass Spectrom. 45, 703-714 2010). The library spectra were filtered in the same manner as the input data. All matches kept between network spectra and library spectra were required to have a score above 0.7 and at least 6 matched peaks. The DEREPLICATOR was used to annotate MS/MS spectra (Mohimani, H. et al. Nat. Commun. 9, 4035 (2018)). The molecular networks were visualized using Cytoscape software (Shannon, P. et al. Genome Res. 13, 2498-2504 (2003)).

References for processing tools:

MZMine: Schmid, R., Heuckeroth, S., Korf, A. et al. Integrative analysis of multimodal mass spectrometry data in MZmine 3. Nature Biotechnology (2023). https://doi.org/10.1038/s41587-023-01690-2

SIRIUS: Kai Dührkop, Markus Fleischauer, Marcus Ludwig, Alexander A. Aksenov, Alexey V. Melnik, Marvin Meusel, Pieter C. Dorrestein, Juho Rousu, and Sebastian Böcker, SIRIUS 4: Turning tandem mass spectra into metabolite structure information. Nature Methods 16, 299–302, 2019.

GNPS: For Feature-Based Molecular Networking: Nothias LF et al. Feature-based Molecular Networking in the GNPS Analysis Environment. bioRxiv 812404 (2019). https://doi.org/10.1101/812404
